# ArdC, a ssDNA-binding protein with a metalloprotease domain, overpasses the recipient *hsdRMS* restriction system broadening conjugation host range

**DOI:** 10.1371/journal.pgen.1008750

**Published:** 2020-04-29

**Authors:** Lorena González-Montes, Irene del Campo, M. Pilar Garcillán-Barcia, Fernando de la Cruz, Gabriel Moncalián

**Affiliations:** Departamento de Biología Molecular, Universidad de Cantabria and Instituto de Biomedicina y Biotecnología de Cantabria (IBBTEC), CSIC-Universidad de Cantabria, Santander, Cantabria, Spain; Uppsala University, SWEDEN

## Abstract

Plasmids, when transferred by conjugation in natural environments, must overpass restriction-modification systems of the recipient cell. We demonstrate that protein ArdC, encoded by broad host range plasmid R388, was required for conjugation from *Escherichia coli* to *Pseudomonas putida*. Expression of *ardC* was required in the recipient cells, but not in the donor cells. Besides, *ardC* was not required for conjugation if the *hsdRMS* system was deleted in *P*. *putida* recipient cells. *ardC* was also required if the *hsdRMS* system was present in *E*. *coli* recipient cells. Thus, ArdC has antirestriction activity against the HsdRMS system and consequently broadens R388 plasmid host range. The crystal structure of ArdC was solved both in the absence and presence of Mn^2+^. ArdC is composed of a non-specific ssDNA binding N-terminal domain and a C-terminal metalloprotease domain, although the metalloprotease activity was not needed for the antirestriction function. We also observed by RNA-seq that ArdC-dependent conjugation triggered an SOS response in the *P*. *putida* recipient cells. Our findings give new insights, and open new questions, into the antirestriction strategies developed by plasmids to counteract bacterial restriction strategies and settle into new hosts.

## Author summary

Horizontal gene transfer is the main mechanism by which bacteria acquire and disseminate new traits, such as antibiotic resistance genes, that allow adaptation and evolution. Here we identified a gene, *ardC*, that enables a plasmid to increase its conjugative host range, and thus positively contributes to plasmid fitness. The crystal structure of the antirestriction protein ArdC revealed a fold different from other antirestriction proteins. Our results have wide implications for understanding how a gene enlarges the environments a plasmid can colonize and point to new targets to harness the bacterial DNA uptake control.

## Introduction

Horizontal gene transfer (HGT) is the transmission of genetic material between organisms that are not in a parent–progeny relationship [1]. The clinical relevance of the HGT process lies in the acquisition and dissemination of genes involved in conferring bacterial resistance to antibiotics (Ab^R^) between unrelated pathogens. When bacteria face selective pressures, as those exerted by antibiotics, horizontal acquisition of Ab^R^ allows the diversification of the genomes, increasing survival opportunities [2]. Conjugation is the main HGT process that allows the transfer of genes encoded in autonomous plasmids. This process requires the machinery to build a direct contact between a donor and a recipient cell [1]. Conjugation can be modulated by environmental factors or bacterial strategies based on genetic approaches that are coded in the chromosome (host barriers) or plasmid DNA (plasmid barriers). Plasmid barriers include entry exclusion [3] or fertility inhibition [4], which reduce conjugative transfer. Host barriers can be mediated through SOS response modulation [5,6], CRISPR-Cas systems [7] or restriction and modification (R-M) systems.

R-M systems allow bacteria to discern between self and foreign DNA invading the cell, leading to its destruction. They require two enzymatic activities: a methyltransferase that provides protection to its own DNA and an endonuclease that cleaves the unmethylated invading DNA [8]. There are four main groups of R-M systems. Type I R-M, the most sophisticated R-M system, requires three genes: *hsdR*, *hsdM*, and *hsdS* and their products associate in R_2_M_2_S complexes. The S subunit recognizes 13–15 bp sequences, usually asymmetric and bipartite. DNA cleavage is at a location away from the specificity site [9–11]. There is a coevolutionary arms race between bacteria to avoid entrance of foreign DNA molecules and parasitic DNA molecules such as plasmids or bacteriophages to enter a putative host avoiding the restriction by bacterial R-M systems. The antirestriction mechanisms to counteract R-M systems can be divided into four main types based on its mode of action: DNA modification, transient occlusion of restriction sites, sabotage of host R-M activities, and inhibition of restriction enzymes [10].

R388 plasmid is the prototype of the IncW incompatibility group of plasmids. IncW plasmids have a low copy number, a wide range of Ab^R^, and a broad host range (BHR) [12]. R388 has 35 genes assorted in functional groups or modules, among them, a gene coding for an antirestriction protein called ArdC [12]. Here, we present ArdC crystal structures and the *ardC* role in interspecies conjugation. We have also identified transcriptional changes associated with *ardC*-mediated conjugation. These results show that ArdC is involved in broadening the R388 plasmid host range.

## Results

### *ardC* is required for R388 conjugation from *E*. *coli* to *P*. *putida*

**Fig 1:**
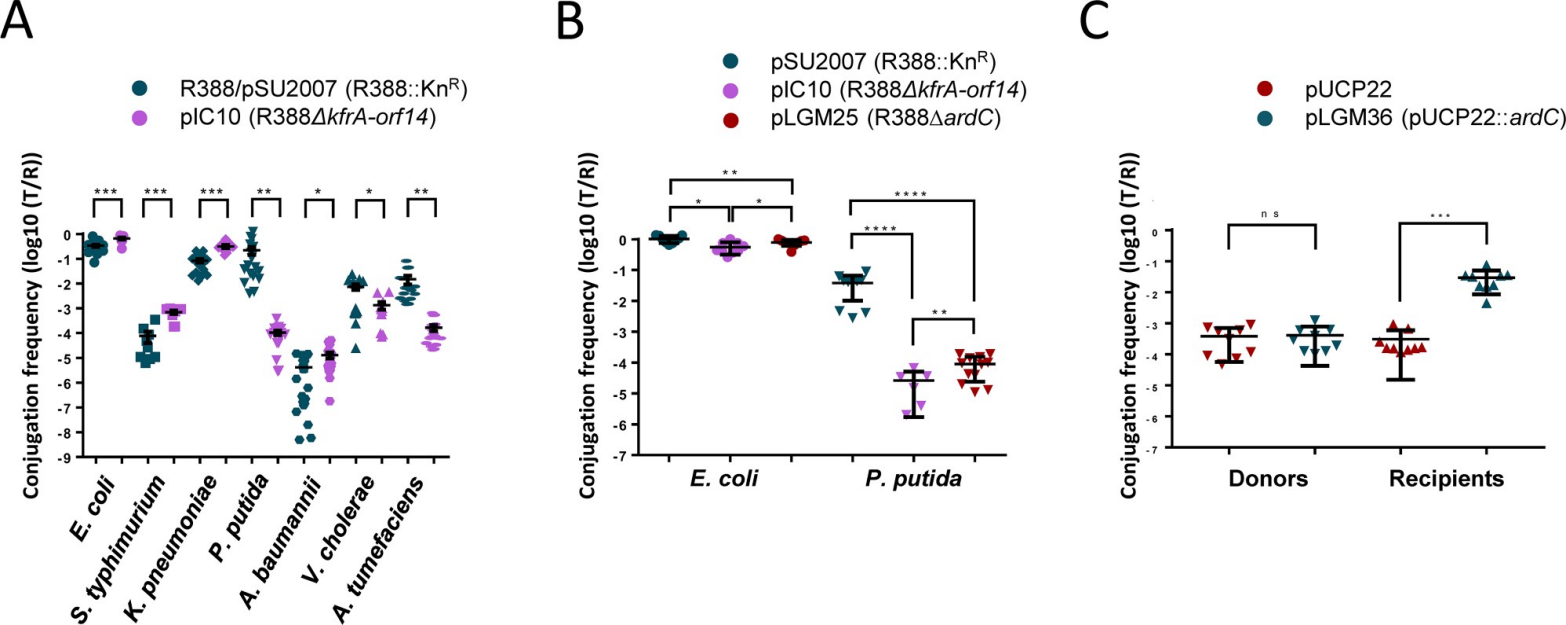
ArdC effect in conjugation. A) Effect of the *kfrA-orf14* region on R388 plasmid conjugative transfer from *E*. *coli* to different bacteria. Conjugations were performed as described in Materials and Methods at 37 ºC except for *P*. *putida* and *A*. *tumefaciens* (done at 30 ºC) for 1 h except for *A*. *baumannii* and *V*. *cholerae* (done for 4 h). R388 was used in conjugations towards *E*. *coli*, *S*. *typhimurium* and *K*. *pneumoniae*. The pSU2007 plasmid was used in conjugations towards the rest of the strains. Donor *E*. *coli* BW27783-RifR cells were used as donors in mating experiments with *E*. *coli*, *S*. *typhimurium*, and *K*. *pneumoniae*. Donor *E*. *coli* BW27783-NxR cells were employed in matings with the rest of the strains. Conjugation frequencies are shown as transconjugants per recipient (T/R). Horizontal bars represent the mean ± SD of N = 9–20 (Student's t-test: * p < 0.1, ** p < 0.01, *** p < 0.001, **** p <0.0001). B) Effect of *ardC* and *kfrA-orf14* deletions on plasmid conjugative transfer (1 h at 37°C) from *E*. *coli* BW27783-Nx^R^ to *E*. *coli* BW27783-Rif^R^ or *P*. *putida* KT2440 (N = 6–12). C) Effect in the conjugation frequency of pLGM25 when expressing *ardC* in donors or recipients. The effect of the presence of plasmid pUCP22 or pUCP22::*ardC* in donors or recipients is shown. Conjugation was done for 1 h at 37°C with 0.1 mM IPTG in the mating mixture (N = 9).

R388 plasmid (GenBank Accession Number BR000038.1) is composed of three functional sectors (S1 Fig): one for general maintenance (modules of replication, stable inheritance and establishment) located in the leading region, a sector for Ab^R^ and integration, and a third one for conjugation (modules of DNA transfer replication and mating pore formation) [12]. We expected the stable inheritance and establishment region to be required in interspecies conjugation. pSU2007, a Kn^R^ R388 derivative, was transferred with different efficiencies from *E*. *coli* BW27783-Nx^R^ to other bacteria (Fig 1A). The transfer of pIC10 (R388Δ*kfrA-orf14)*, an R388 derivative without the stability and maintenance region, was more dissimilar to that of pSU2007 from *E*. *coli* to *P*. *putida* KT2440 where the conjugation frequency dropped around 1000-fold. In the stability and maintenance gene region deleted in pIC10 there are 13 genes that code for proteins homologous to some with predicted function of fertility inhibition (*osa*) [13], proteins of unknown function (*klcB*, *nuc1*, *nuc2*, *orf7*, *orf8*, *orf9*, *orf12*, and *orf14*), transcriptional regulators (*kfrA*, and *ardK*), ssDNA binding protein (*ssb*), and antirestriction (*ardC*). ArdC protein (297 amino acids and 33.2 KDa, GenBank Acc. No. FAA00054.1) exhibited an *in vitro* antirestriction function towards Type I and II R-M systems [14]. Thus, we constructed plasmid pLGM25 (R388Δ*ardC*) to check if the effect observed in conjugation with pIC10 could be due to the lack of the *ardC* gene. This plasmid was introduced into *E*. *coli* BW27783-Nx^R^ and then conjugated to *E*. *coli* BW27783-Rif^R^ or *P*. *putida* KT2440 (Fig 1B*)*. We observed that the absence of *ardC* in the conjugative plasmid pLGM25 reduced the conjugation frequency to *P*. *putida* from 3.8E-02 to 9.0E-05, but not to *E*. *coli*. Thus, the results observed for pIC10 could be explained to a large extent by *ardC* absence.

### *ardC* is needed in recipient cells

To check if ArdC was needed in donor or recipient cells, transfer of Δ*ardC* pLGM25 plasmid was measured when complemented by the overexpression of *ardC* in donor *E*. *coli* cells, or in recipient *P*. *putida* cells. As shown in Fig 1C, *ardC* did not improve the conjugation frequency when overexpressed in donors. On the other hand, overexpression of *ardC* in recipient cells increased the conjugation frequencies, reaching pSU2007 conjugation levels. Thus, it seems that the expression of ArdC is specifically required in the recipient, and not in donor cells.

### ArdC is a ssDNA-binding protein with a metalloprotease domain

R388 ArdC crystal structure was solved at 2.6 Å resolution using a selenomethionine-derivative protein structure solved by single anomalous dispersion as described in Materials and Methods. Using this preliminary structure, the apo ArdC structure was solved at 2.0 Å resolution by molecular replacement (MR). Apo ArdC crystallized in the H3 space group containing one molecule per asymmetric unit. Data collection and refinement statistics are given in Table 1.

**Table 1:** Data collection and refinement statistics for ArdC structures ^a^.

	ArdC SeMet	ArdC native	ArdC-Mn
**Wavelength**	(peak)	0.9792	0.9793
**Resolution range**	47–2.6 (2.69–2.6)	39.5–2.0 (2.07–2.0)	54.8–2.7 (2.8–2.7)
**Space group**	R 3: H	R 3: H	P 32
**Unit cell**	a = b = 136.9 c = 51.3 α = β = 90 γ = 120	a = b = 136.8 c = 51.7 α = β = 90 γ = 120	a = b = 116.5 c = 162.1 α = β = 90 γ = 120
**Total reflections**	443798 (44597)	537291 (33696)	1158240 (114678)
**Unique reflections**	10970 (1098)	24366 (2408)	66345 (6492)
**Multiplicity**	40.5 (40.6)	22.1 (13.9)	17.5 (17.4)
**Completeness (%)**	99.82 (99.64)	99.8 (98.5)	96.6 (96.9)
**Mean I/sigma(I)**	66.78 (6.54)	37.1 (3.1)	25.7 (2.6)
**Wilson B-factor**	46.69	31.87	47.81
**R-merge**	0.5962 (1.055)	0.7636 (1.274)	0.6992 (1.47)
**R-meas**	0.6046 (1.07)	0.7814 (1.326)	0.7198 (1.514)
**CC1/2**	0.937 (0.901)	0.773 (0.474)	0.691 (0.572)
CC*	0.984 (0.974)	0.934 (0.802)	0.904 (0.853)
**Reflections used in refinement**		24339 (2406)	65278 (6491)
**Reflections used for R-free**		1230 (111)	3008 (320)
**R-work**		0.1726 (0.2154)	0.2207 (0.2823)
**R-free**		0.1976 (0.2410)	0.2943 (0.3499)
**CC(work)**		0.864 (0.814)	0.855 (0.672)
**CC(free)**		0.817 (0.837)	0.845 (0.581)
**Number of non-hydrogen atoms**		2412	16374
**Protein residues**		276	2008
**RMS(bonds)**		0.008	0.010
**RMS(angles)**		1.16	1.17
**Ramachandran favored (%)**		97.76	92.79
**Ramachandran allowed (%)**		1.49	5.71
**Ramachandran outliers (%)**		0.75	1.5
**Rotamer outliers (%)**		0.00	0.00
**Clashscore**		3.46	16.63
**Average B-factor**		36.39	46.75

^a^ Statistics for the highest-resolution shell are shown in parentheses.

**Fig 2:**
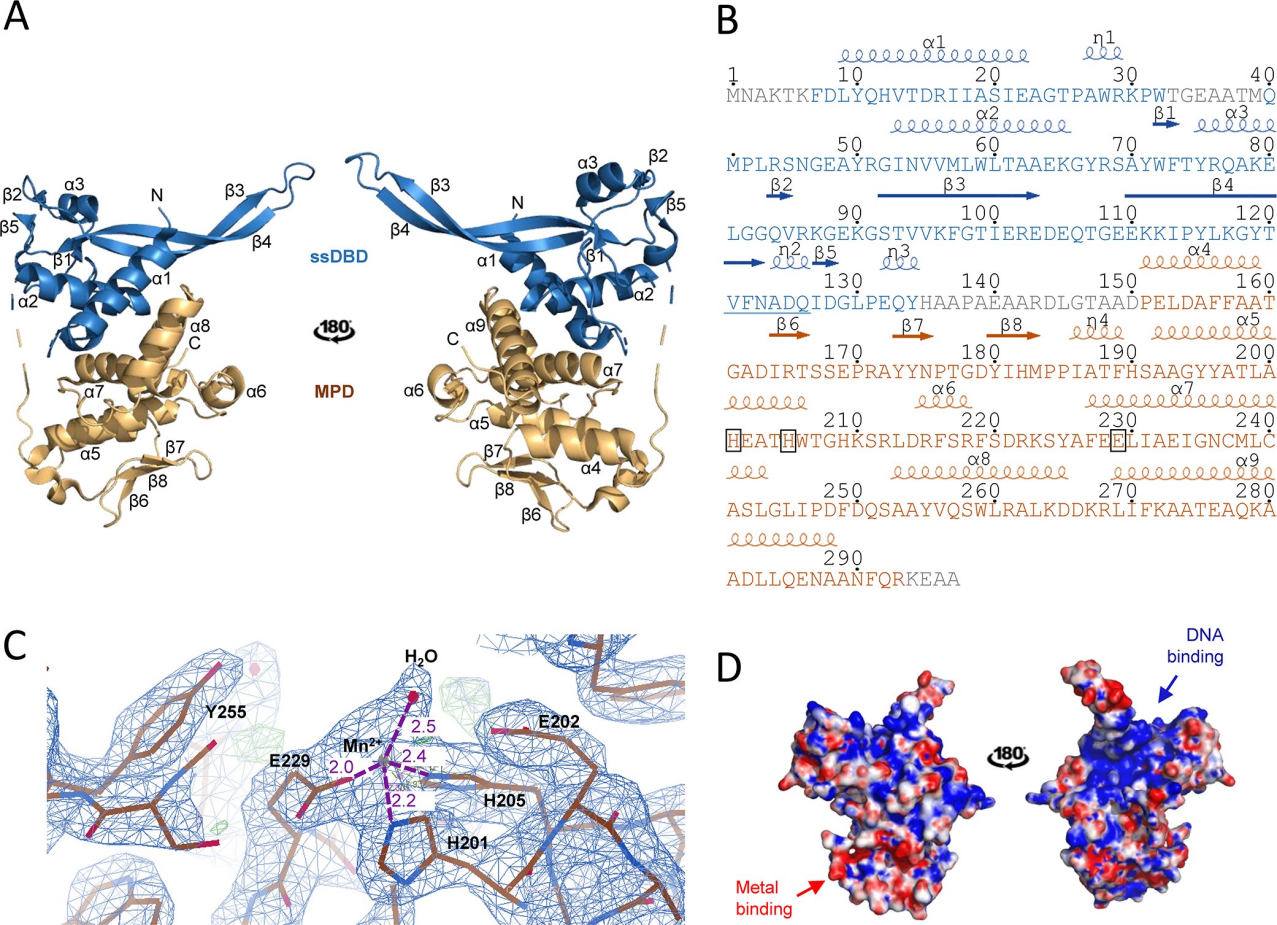
ArdC structure. A) Cartoon representation of two views of the ArdC structure. N-terminal ssDNA-binding domain (ssDBD) is shown in blue and C-terminal metalloprotease domain (MPD) in orange. α-helices are labeled from α1 to α9 and β-strands are labeled from β1 to β8. A dashed line schematizes the disordered loop joining both domains. B) ArdC sequence with secondary structure information. ArdC sequence is colored by domains and α-helices and β-strands are labeled as in A). 3_10_ helices are labeled from η1 to η3. The residues involved in metal coordination are framed. The “squiggle” signature proposed by [15] for Rad4 is underlined in blue. C) Electron density of the metal-binding site in the ArdC-Mn crystal structure solved at 2.7 Å resolution. Residues and molecules involved in metal coordination (H201, H205, E229, and H_2_O) or activity are labeled. Distance in Å to the metal is shown in purple. D) Electrostatic potential surface. The negative surface is colored in red and the positive surface in blue (calculated by APBS tool). The expected binding areas for DNA and metal cofactor are indicated.

ArdC is composed of two structural domains: An N-terminal domain (residues 1–134) and a C-terminal domain (residues 151–297) joined by a long and flexible loop (135–150) (Fig 2A and 2B). Electron density was not observed for the N-terminal residues 1–6, the flexible small loop residues 33–39, residues 136–141 in the region connecting both domains and C-terminal residues 294–297. The N-terminal domain is composed of three α-helices (α1-α3), a three-stranded β-sheet (β1, β3, and β4) that supports a long and protuberant β-hairpin (β3-β4), a smaller two-stranded antiparallel β-sheet formed by β2 and β5, as well as three 3_10_ helices labeled from η1 to η3 (Fig 2A and 2B). The C-terminal domain is composed of six α-helices (α4-α9) and short three-stranded antiparallel β-sheets (β6-β8) as shown in Fig 2A and 2B.

**Fig 3:**
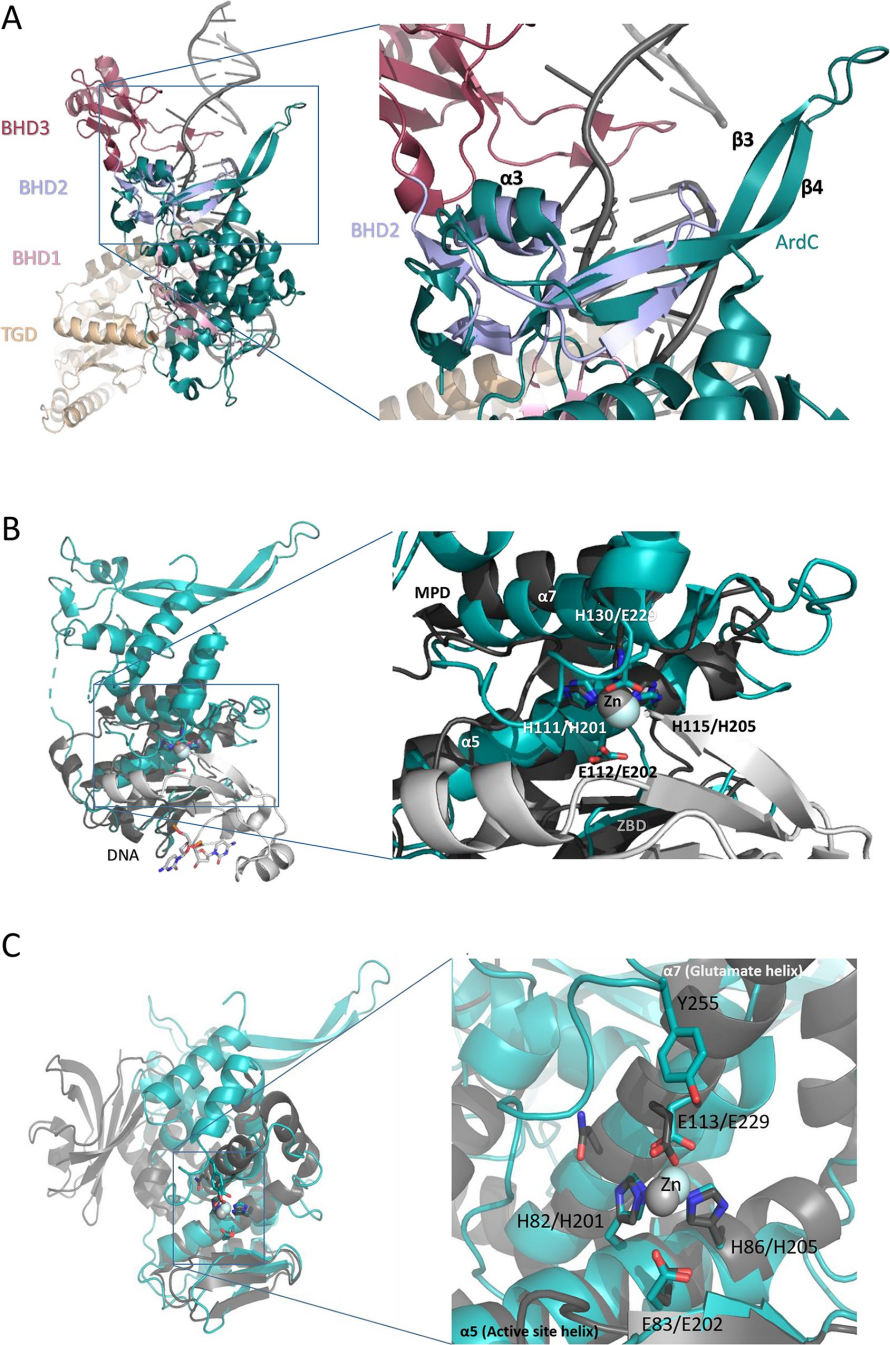
ArdC structural homologs. A) Superposition of ArdC (green) with the Rad4 protein bound to UV-damaged DNA (2QSG). The Rad4 TGD domain is shown in beige, BHD1 in pink, BHD2 in purple, and BHD3 in red. For clarity, Rad23 (present in 2QSG structure) has been removed. Detailed view of ArdC ssDBD domain superposed to the Rad4 BHD2 domain. B) Superposition of ArdC (green) with the Spartan SprT domain (6MDX; grey). The SprT Zn2+-binding sub-domain (ZBD) is shown in light grey and the metalloprotease sub-domain (MPD) is shown in dark grey. Detailed view of the metalloprotease active center with the residues involved in catalysis in sticks numbered as (MPD/ArdC). C) Superposition of the ArdC structure (green) with the IrrE-Zn protein from *Deinococcus radiodurans* (3DTI, grey). Detailed view of the active center with the residues involved in catalysis in sticks numbered as (IrrE/ArdC).

The ArdC structure was compared to those deposited in the PDB using the Dali server [16]. ArdC N-terminal domain closest structural homolog is present in a nucleotide excision repair protein called Rad4 (PDB: 2QSG; Z-score: 4.5), a component of the eukaryotic nucleotide excision repair (NER) pathway. Rad4 is composed of an inactive transglutaminase fold domain and three different β-hairpin domains (BHD). The three tandem BHD domains form a large DNA binding surface [17]. ArdC N-terminal domain is more similar at a sequence level to the second BHD domain of Rad4 (BHD2), although BHD2 is considerable smaller (about 50 amino acids long compared to the 134 residues of ArdC-N). It lacks some ArdC-N structural features, such as the starter ArdC α1, α2, and final 3_10_ motifs. Moreover, the protuberant β-hairpin formed by β3 and β4 is larger in ArdC (Fig 3A). ArdC N-terminal domain possesses the V^121^FNADQ^126^ sequence located within a 3_10_ helix (η2) between β4 and β5 (Fig 2B). This region forms a crossover with the β2 to β3 region and creates a sharp twist of the chain known as the “squiggle” motif [15]. This motif in Rad4 is proposed to be responsible for a highly flexible region that could facilitate recognition of DNA sequences.

**Fig 4:**
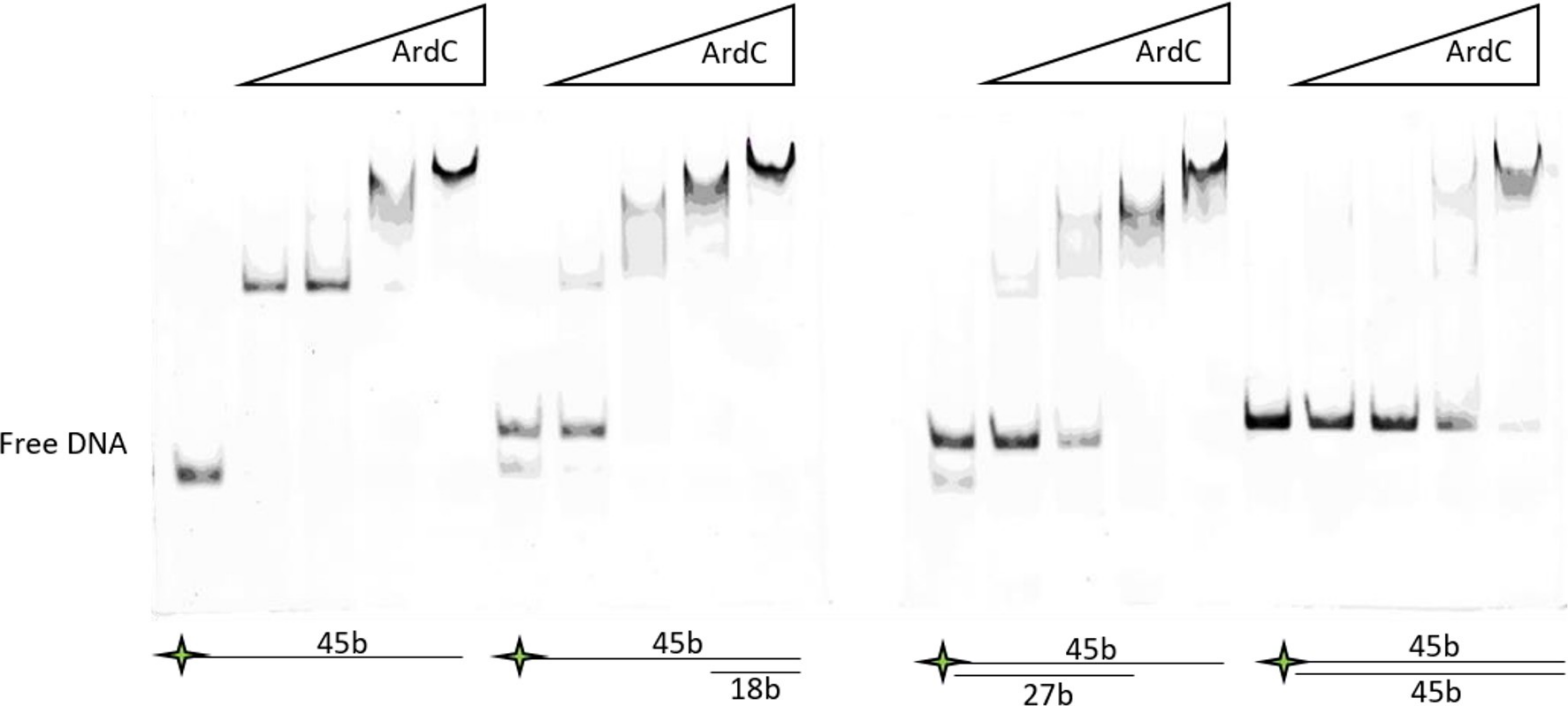
ArdC DNA-binding preferences assessed by EMSA. ArdC binding of a 6FAM-labeled 45 bases ssDNA oligonucleotide (Fluor-T87I2), a perfectly paired complementary 45bp dsDNA duplex (Fluor-T87I2 + T87I1), and two partial dsDNA with 5’ or 3’ terminal ssDNA overhangs (Fluor-T87I2 + Mid1) and (Fluor-T87I2 + Mid2) was performed at increasing concentrations of ArdC (0, 125 nM, 250 nM, 500 nM, and 1 μM), as described in Materials and Methods. Protein-DNA complexes were resolved by native 10% polyacrylamide gels and visualized by a fluorescent image analyzer.

The surface electrostatic map (Fig 2D) reveals a positively charged groove in the region of the N-terminal domain adjacent to the C-terminal domain, suggesting a DNA binding site between both structural domains. By electrophoretic mobility shift assays (EMSA), we determined that ArdC preferentially binds ssDNA oligonucleotides over dsDNA molecules (Fig 4) in accordance with previous results [14]. Moreover, binding to partial dsDNA with 5’ or 3’ terminal ssDNA overhangs is preferred over binding to perfectly paired complementary dsDNA duplex (Fig 4). We will name ArdC N-terminal domain hereafter ssDNA-binding domain (ssDBD).

Regarding the C-terminal domain (amino acids 151–297), according to the Dali server [16] the closest structural homologs were the DNA binding metalloproteases Spartan (PDB: 6MDW; Z-score: 6.4) and IrrE (PDB: 3DTE; Z-score: 5.5) (Fig 3C and 3D). Spartan is a protein involved in the cleavage of proteins irreversibly cross-linked to DNA to preserve this way genome stability [18]. IrrE protects *D*. *radiodurans* from UV radiation DNA damage by proteolysis of a negative transcriptional regulator, DdrO, which represses the expression of DNA damage response genes involved in SOS response [19]. These proteins belong to the gluzinzin metalloprotease family characterized by the presence of the conserved residues HExxH located on the “active site helix” (α5 in ArdC) and an additional conserved motif (E,H)xx(A,F,T,S,G) located in the contiguous α-helix or “glutamate helix” (α7 in ArdC) [20] (Fig 2A and 2B). The surface electrostatic map revealed a negatively charged catalytic pocket (Fig 2D). We will name ArdC C-terminal metalloprotease domain MPD.

The two histidines of the active site helix and the glutamic acid of the glutamate helix have been shown to coordinate a catalytic divalent metal ion, usually zinc. However, gluzinzin metalloproteases maintain the catalytic activity with Co^2+^, Mn^2+^ or Ni^2+^ too due to the flexibility of these three metal coordination geometries [20,21]. To assess the metal used by ArdC the stability of ArdC was assayed in the presence of different metal cofactors. ArdC showed increased thermal stability (assayed by ThermoFluor) in the presence of Ni^2+^, Mn^2+^ or Co^2+^ (ΔT_M_> 4°C), but not in the presence of Zn^2+^, Ca^2+^, Mg^2+^, Cu^2+^ or Fe^3+^ (S1 Table). Moreover, to know the conformation of the active site when bound to metals, ArdC was crystallized in the presence of MnCl_2_ (Materials and Methods). ArdC-Mn crystallized in the P32 space group containing eight molecules per asymmetric unit and the structure was solved at 2.7 Å (Table 1). Mn^2+^ is tetrahedrally coordinated by H201, H205, E229 and an H_2_O molecule (Fig 2C). H205 is oriented towards the metal by interaction with the conserved E228 through the non-coordinating nitrogen atom. The E202 residue of the HE^202^xxH motif orients and acts as a catalytic base for the activation of a water molecule that coordinates the metal. The H_2_O molecule could act as a Lewis acid to allow the nucleophilic attack [20]. By analogy with other gluzinzin metalloproteases, the conserved ArdC residue Y255 could stabilize by a hydrogen bond the polypeptide chain to be cleaved [22]. The metalloprotease sub-domain (MPsD) in Spartan shares the active center structure with ArdC MPD except that MPsD uses a third histidine instead of a glutamic acid for metal coordination (Fig 3B).

It had been proposed that ArdC could avoid ssDNA degradation by HhaI, a type II restriction enzyme able to cleave both ssDNA and dsDNA [14]. According to our structural results, this ArdC DNA protection could be due to ArdC MPD activity targeting the restriction enzyme. To test this hypothesis, the inhibition of HhaI by ArdC was assayed in the presence of ssDNA M13mp18 (7.2 kb) and Mg^2+^. As observed in S2 Fig, ArdC was able to avoid ssDNA cleavage by HhaI but we did not observe HhaI degradation by ArdC.

Since its 3D structure defined ArdC as a protease, we tried to find a specific protein target. ArdC mutant E229A (supposed to be inactive) was purified and used as prey for co-purification of potential targets in *P*. *putida* KT2440 cell lysate by the pull-down technique. The only protein that co-eluted with ArdC was PP_0941, a protein of unknown function similar to the 50S ribosome subunit associated protein YjgA (S3 Fig).

### SOS response is activated in *P*. *putida* recipient cells by the transfer of an *ardC*-containing plasmid

IrrE, the bacterial closest structural homolog to ArdC, triggers SOS response by cleaving the transcriptional regulator DdrO in an analogous way to the RecA-LexA system [19]. To check if ArdC could have similar activity on plasmid conjugation, we analyzed by RNA-seq changes in gene expression when an a*rdC*-containing plasmid was transferred from *E*. *coli* to *P*. *putida*. As described in Materials and Methods, *P*. *putida* KT2440 was mixed in a conjugation filter with either *E*. *coli* BW27783-Nx^R^ bearing no plasmid (NP), *E*. *coli* BW27783-Nx^R^ bearing pSU2007 (*ardC*^*+*^) or *E*. *coli* BW27783-Nx^R^ bearing pLGM25 (*ardC*^*-*^).

As expected according to the results shown in Fig 1B, significant conjugation frequency differences between *ardC*^*+*^ and *ardC*^*−*^conditions were observed (S2 Table). RNA-seq results (S3–S5 Tables and S4 Fig) showed that: (a) R388 genes involved in conjugation are highly upregulated in the *ardC*^*+*^ condition regarding the *ardC*^*−*^condition (S5 and S6 Tables and S4A Fig). This is consistent with the zygotic induction observed in the recipient cells after conjugation [23]; (b) several donor *E*. *coli* genes and pathways involved in flagellar motility, SOS and stress responses and different metabolic pathways are downregulated in the *ardC*^*−*^condition regarding the NP or *ardC*^*+*^ conditions (S5 and S7 Tables, and S4B Fig); (c) SOS genes are upregulated in recipient *P*. *putida* cells when receiving *ardC*^*+*^ containing plasmid with respect to NP or *ardC*^*−*^conditions. Differential expression of *P*. *putida* genes in recipient cells when the ardC-containing plasmid is transferred is shown in S8 Table and S4C Fig.

### ArdC metalloprotease activity is not required for conjugation to *P*. *putida*

**Fig 5:**
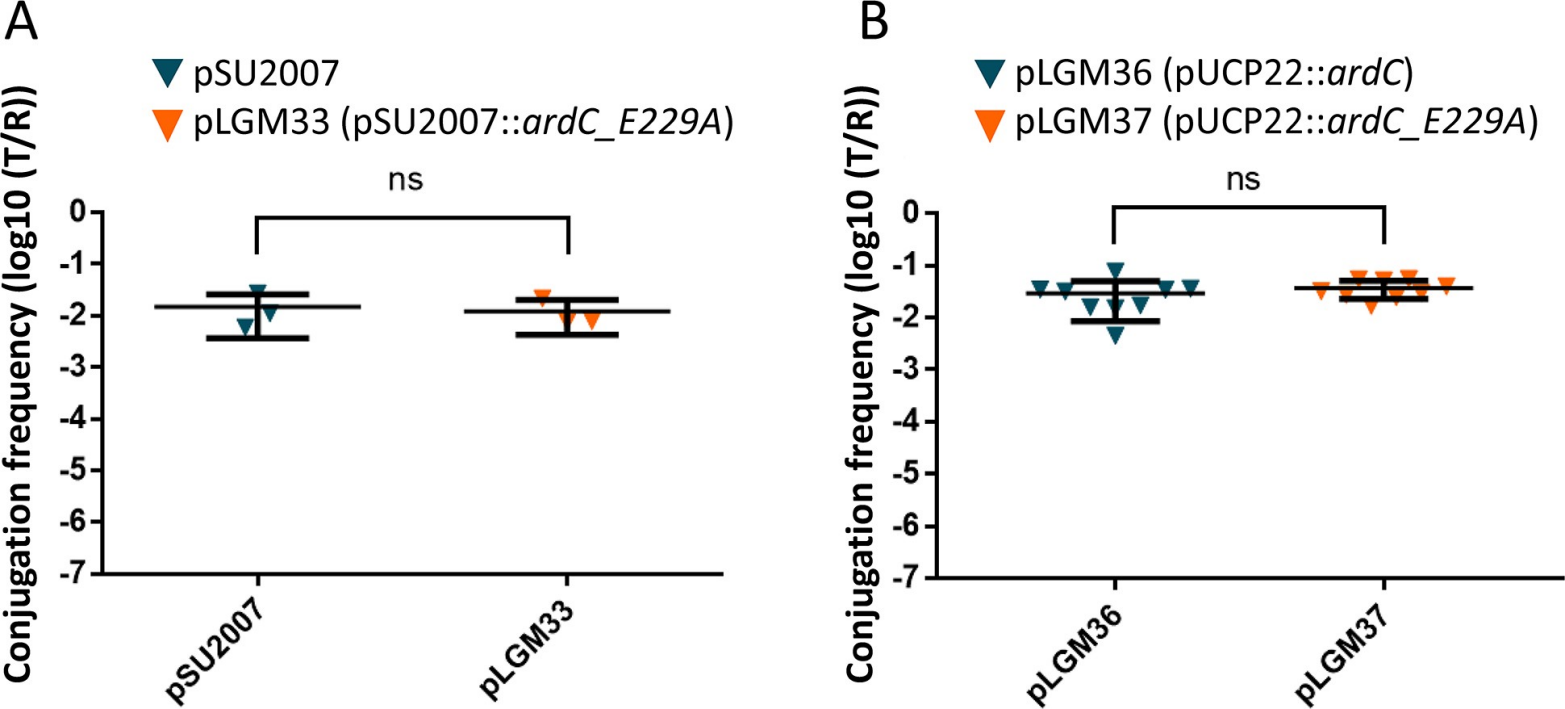
Effect of ArdC E229A mutant on plasmid conjugative transfer. A) Conjugation of *E*. *coli* BW27783 bearing pSU2007 or pLGM33 (pSU2007_*ardC_E229A*) to *P*. *putida* KT2440. Conjugation was performed for 1 h at 37°C. Horizontal bars represent the mean ± SD of N = 3 observations. B) Effect in the conjugation frequency of pLGM25 when expressing *ardC_E229A* in recipient cells. Conjugation of pLGM25 in *E*. *coli* BW27783 donor cells to *P*. *putida* KT2440 recipient cells bearing pUCP22::*ardC* or pUCP22:: *ardC_E229A*. Conjugation was performed for 1 h at 37°C with 0.1 mM IPTG added to the mating mixture (N = 9).

Being ArdC a ssDNA binding protein with a metalloprotease domain, we checked if this proteolytic activity was needed for ArdC activity in conjugation. The *ardC* gene was mutated to *ardC_E229A* in pSU2007 to generate plasmid pLGM33. Mutation of the glutamic acid of the active site involved in metal coordination to alanine, E229A, is expected to deactivate the proteolytic center of ArdC, as it occurs in other family members. Plasmids pSU2007 or pLGM33 were conjugated from *E*. *coli* to *P*. *putida*. Surprisingly, pLGM33 conjugation frequency resulted to be like that of pSU2007, around 0.01 transconjugants per recipient (T/R) (Fig 5A). Besides, the *ardC_E229A* mutant gene was tested for its ability to complement pLGM25 (R388 *ΔardC*) plasmid in mating experiments from *E*. *coli* to *P*. *putida* KT2440. The *in trans* expression of *ardC_E229A* in *P*. *putida* KT2440 recipient cells was able to increase the conjugation frequency of pLGM25 at the same levels as the expression of the wt *ardC* gene (Fig 5B). Thus, ArdC metalloprotease activity is not required for the host range broadening activity at least in *P*. *putida*.

### ArdC counteracts the HsdRMS system in both *P*. *putida* and *E*. *coli*

**Fig 6:**
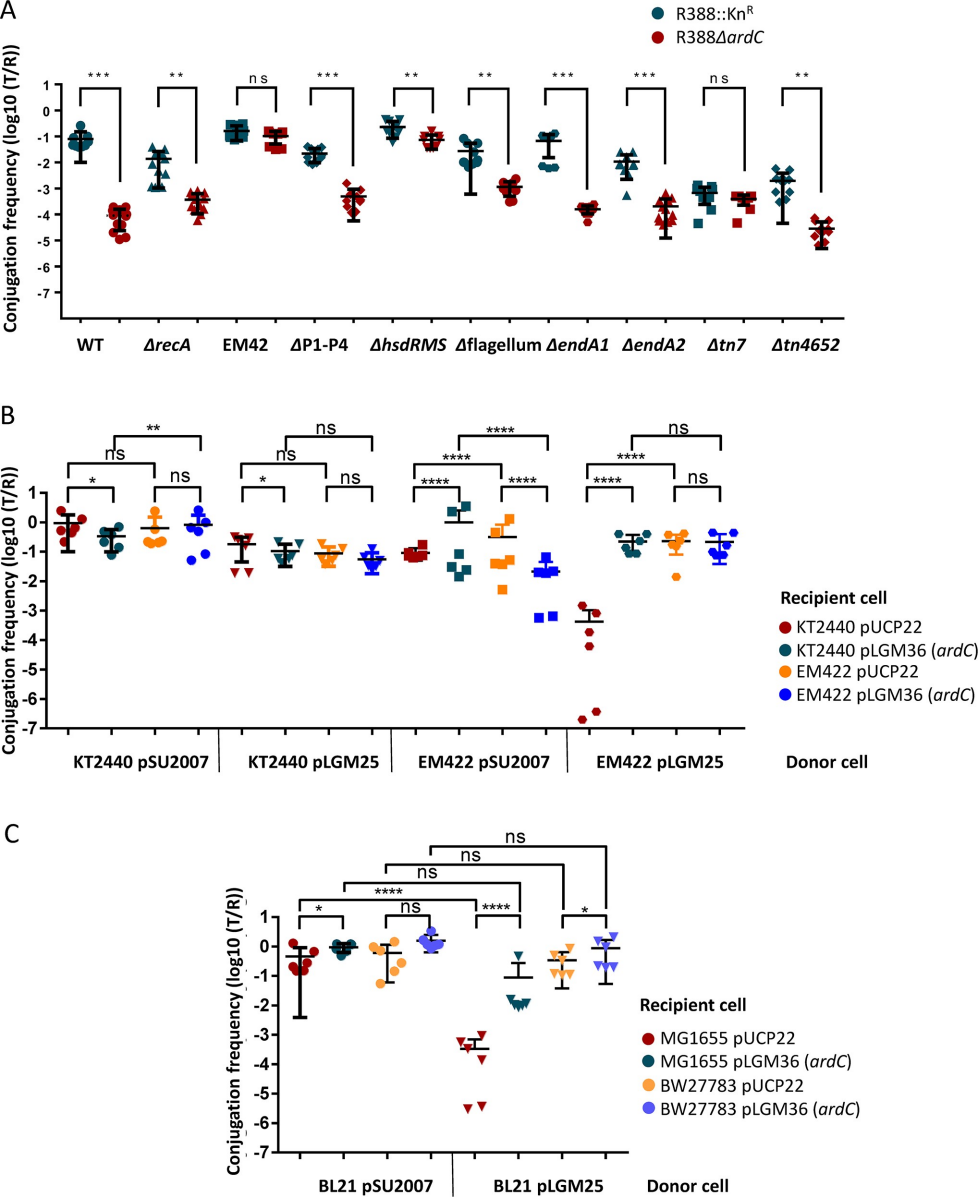
Effect of HsdRMS systems on R388 plasmid conjugation. A) Effect of ArdC on plasmid conjugative transfer from *E*. *coli* to *P*. *putida* KT2440 mutants. The conjugation frequencies (T/R) to *P*. *putida* KT2440 wt strain or different mutants were obtained after conjugation for 1h at 37°C. The deleted gene(s) in each strain is shown. EM42 is Δprophage1, Δprophage4, Δprophage3, Δprophage2, Δ*tn7*, Δ*endA-1*, Δ*endA-2*, Δ*hsdRMS*, Δflagellum, and Δ*tn4652*. ΔP1-P4 stands for Δprophage1 Δprophage4 Δprophage3 Δprophage2 strain. Horizontal bars represent the mean ± SD obtained for each dataset of N = 8–12 (Student's t-test: * p < 0.1,** p < 0.01, *** p < 0.001, **** p <0.0001). The effect of *ardC* in the transfer of T-DNA methylated or not to restricting or non-restricting *P*. *putida* B) or *E*. *coli* recipients C) is evaluated in mating experiments performed as in A with N = 6.

To identify the functional target of ArdC, different *P*. *putida* mutant strains were assessed as recipients in mating experiments. RecA dependent SOS response is activated in *ardC*^*+*^ conjugation recipient cells as shown by the RNA-seq experiments (S8 Table). To check the role of this response in conjugation, firstly pSU2007 and pLGM25 were conjugated from *E*. *coli* to *P*. *putida* KT2440*ΔrecA*. The frequency of conjugation of pSU2007 to *P*. *putida* KT2440*ΔrecA* was about 10^−2^ T/R (Fig 6A). Thus, RecA dependent SOS response in *P*. *putida* recipient cells is not essential for conjugation. Moreover, the frequency of conjugation of pLGM25 to *P*. *putida* KT2440*ΔrecA* was around 10^−4^ T/R, meaning that the absence of *recA* does not enhance conjugation in the absence of *ardC*. In addition, pSU2007 and pLGM25 were conjugated from *E*. *coli* to *P*. *putida* EM42, which carries deletions of several prophages and other accessory genes (*Δ*prophage1 *Δ*prophage4 *Δ*prophage3 *Δ*prophage2 *Δtn7 ΔendA-1 ΔendA-2 ΔhsdRMS Δ*flagellum *Δtn4652*) that could harass the heterologous gene expression (because their association to genetic instability or attributed to the unfruitful usage of metabolic resources). Any of these genes removed from *P*. *putida* EM42 could affect the establishment of the plasmids acquired by conjugation. The conjugation frequency towards EM42 strain was not affected by the *ardC* deletion (Fig 6A) indicating that ArdC could be counteracting the action of the products of one or more of the deleted genes in the EM42 strain. To identify the gene(s) responsible for the observed phenotype, pSU2007 and pLGM25 were conjugated from *E*. *coli* to *P*. *putida* mutants with deletions in single or several genes. pLGM25 only reached pSU2007 conjugation levels (around 0.1 T/R) in the *P*. *putida* KT2449*ΔhsdRMS* strain EM422 (Fig 6A). Similar results were observed at 30°C (S5 Fig). The *hsdRMS* operon was thus the main responsible for the effect observed in EM42. pLGM25 was efficiently transferred between *P*. *putida* KT2440, being its conjugation frequency around 0.1 T/R (Fig 6B). However, pLGM25 transfer drastically dropped from *P*. *putida* EM422 (*ΔhsdRMS*) to *P*. *putida* KT2440 unless pLGM36 was present in the *P*. *putida* KT2440 recipient cell. Thus, *ardC* is counteracting the effect of the HsdRMS R-M system in the incoming DNA.

The *E*. *coli* strain BW27783 used as donor and recipient in the mating experiments shown in Fig 1B was a non-restricting and modifying strain (r_K_^-^m_K_^+^), and thus not suitable to check for the ArdC effect in R388 conjugation between *E*. *coli* cells. So, to evaluate if the *E*. *coli* HsdRMS R-M system was also targeted by ArdC, a non-restricting and non-modifying (r_B_^-^m_B_^-^) *E*. *coli* strain, BL21(DE3), was used as a donor. When pLGM25 was transferred to *E*. *coli* MG1655 (r_K_^+^m_K_^+^) its conjugation frequency significantly decreased in comparison to pSU2007 transfer, but not to *E*. *coli* BW27783 (r_K_^-^m_K_^+^) (Fig 6C). Moreover, the conjugation frequency was again rescued when ArdC was expressed in the recipient cell.

## Discussion

Antibiotic resistance determinants and xenobiotic degradation genes are extensively disseminated in different ecological niches by conjugative BHR plasmids. BHR plasmids evolved different strategies to avoid obstacles to their entrance in new recipient cells. In this article, we determined that ArdC protein, produced by the IncW BHR plasmid R388, is required for interspecies conjugation from *E*. *coli* to *P*. *putida*. ArdC was first studied by [14], who showed an *in vitro* antirestriction function towards Type I and II R-M systems. They observed that ArdC showed a 38% identity with the N-terminal region (about 300 amino acids, DUF1738 domain (pfam08401)) of TraC1 primase from RP4 plasmid. Since TraC1 travels to the recipient cell during conjugation presumably bound to the ssDNA that is being transferred (T-strand) [14], they proposed that ArdC could be also transferred during conjugation bound to the plasmid T-strand. Besides, they proposed that ArdC protects the incoming DNA from host endonucleases through restriction site occlusion. However, they failed to detect any significant influence of ArdC on the efficiency of an IncW plasmid transfer between a non-restricting and non-modifying donor *E*. *coli* and an EcoK-restricting recipient *E*. *coli* strain. We demonstrated in this work that ArdC plays indeed an *in vivo* function in IncW plasmid conjugation. ArdC role involves its expression in recipient cells. Consequently, complementation of pLGM25 (R388Δ*ardC*) with *ardC* in donor cells neither recovered wt plasmid conjugation frequencies from *E*. *coli* to *P*. *putida* nor between *E*. *coli* strains, while ArdC expression in the recipient cells did. Thus, although our experiments do not rule out ArdC export during conjugation in the wild type system, it is not a requirement for its activity, contrary to what was proposed [14]. TraC1 of RP4 and Sog primases of IncI1 plasmids are transferred to the recipient cell during conjugation[24]. They have in common a topoisomerase-primase (TOPRIM) domain located either in the C-terminal part of TraC1 or the N-terminal part of Sog. Both primases differ in that TraC1 contains the DUF1738 domain, which is also present in ArdC. TOPRIM is not present in ArdC. The antirestriction activity of the TraC1 primase has not been investigated but, in the light of our results is a reasonable hypothesis. In any case, a putative TraC1 antirestriction activity seems redundant, since antirestriction against the Type I R-M system was demonstrated both *in vivo* [25] and *in vitro* [26] for KlcA, another RP4 protein.

DUF1738 and TOPRIM domain accretion in a single protein seems to be a specific adaptation. Inspection of the RefSeq plasmid database (version 90) using Hidden Markov Model searches for TOPRIM (PF13362) and DUF1738 (PF08401) domains revealed the presence of 1,334 TOPRIM-containing proteins and 877 DUF1738-containing proteins. Only 84 plasmid proteins contained both domains and they were encoded in 83 plasmids. Additionally, 203 plasmids encoded both domains, but each in a separate protein thus totalling 286 plasmids encoding both DUF1738 and TOPRIM domains, either fused or independent (S6 Fig). An overwhelming proportion of plasmids encoding either DUF1738 or TOPRIM domains also encoded a MOB relaxase and can thus be considered transmissible by conjugation [27]: 939 out of 1242 TOPRIM-encoding plasmids, 667 out of 831 DUF1738-encoding plasmids, 252 out of 286 DUF1738+TOPRIM-encoding plasmids, and 82 out of 83 plasmids encoding both domains in the same protein. While transmissible plasmids encoding TOPRIM and DUF1738 domains in separate proteins were distributed in seven different MOB classes, all but one of those encoding both domains in the same polypeptide belonged to the MOB_P_ class (roughly half of them IncP1 plasmids).

Further information about ArdC activity was obtained by solving its crystal structure. Interestingly, ArdC contains an N-terminal ssDNA binding domain and a C-terminal metalloprotease domain. ArdC is structurally similar to DNA-binding dependent metalloproteases involved in the maintenance of genetic stability such as Spartan or IrrE. Human Spartan protein cleaves DNA-protein crosslinks [18], while IrrE plays a central regulatory role in DNA protection and repair pathways in response to radiation [19]. ArdC structure differs from other known plasmid-encoded antirestriction proteins. ArdA from the conjugative transposon Tn*916* (2W82) is structurally similar to the B-form DNA, this way binding Type I R-M systems to avoid DNA degradation [28]. KlcA (PDB:2KMG) from the IncP-1β plasmid pBP136 and ArdB (PDB:2WJ9) are composed of a single α/β domain inhibiting the endonuclease activity of Type I R-M systems by an indirect mechanism not related to the mimic of DNA structure [29]. According to our solved structure, we expect ArdC to provide a new antirestriction mechanism.

We observed SOS response activation in *P*. *putida* recipient cells during *ardC*^*+*^-mediated conjugation from *E*. *coli*. ArdC could trigger SOS response in recipient cells similarly than IrrE triggers SOS response upon radiation damage (by proteolysis of DdrO, a transcriptional regulator involved in SOS response) [30]. However, it has also been described that conjugative ssDNA activates SOS response when the plasmid cannot replicate in the recipient [31]. As ArdC_E229A mutant is still able to promote R388 conjugation to *P*. *putida*, we think that activation of the SOS response in the recipient is the consequence of the ongoing conjugative process and not a direct effect of the presumed proteolytic activity of ArdC. We hypothesize that the high affinity of ArdC by ssDNA could delay the complementary strand replication resulting in a transient higher amount of ssDNA and thus triggering the SOS response. This is supported by the fact that pLGM25 transfer to *P*. *putida* did not result in SOS activation.

Since ArdC is not required for R388 conjugation either to *P*. *putida* KT2440Δ*hsdRMS* or to *E*. *coli* Δ*hsdR*, it is expected to play a role as counteracting Type I R-M system, probably preventing degradation of the transferred DNA. Moreover, ArdC is not required for R388 conjugation between *P*. *putida* KT2440 strains. Consequently, once R388 is modified by the HsdRMS system, it can be properly transferred to another cell also containing the same HsdRMS system. Type I R-M systems attack dsDNA and thus are not expected to degrade ssDNA during bacterial conjugation. However, it has been reported that the EcoKI R-M system affects the uptake of DNA by conjugation [32] and, ArdC is not expressed until plasmid DNA is in dsDNA shape. Thus, Type I R-M system could be attacking late, once ArdC is generated from the dsDNA plasmid after DNA entrance and establishment in recipient cells. In this respect, the observation that mutation of the ArdC metalloprotease active center does not reduce interspecies conjugation suggests that the metalloprotease activity is not required during conjugative transfer to *P*. *putida*. The presumed metalloprotease activity is not expected to play a role in HsdRMS activity. Thus, it is tempting to propose that, just by ArdC binding to DNA, the protein interferes with HsdRMS binding and thereby hindering the degradation of its target DNA.

In summary, our results indicate a new mechanism of DNA antirestriction played by protein ArdC, by which plasmids increase their conjugation host range. Interfering with ArdC activity could thus provide a new tool to hinder the transmission of antibiotic resistance.

## Materials and methods

### Conjugation assays

Conjugation assays were performed by mixing *E*. *coli* or *P*. *putida* cells (Table 2) containing the plasmid of interest (Table 3) with recipient cells grown overnight at their optimal growing temperature. *E*. *coli* cells were grown at 37 ºC and *P*. *putida* at 30 ºC. Cells at OD600 = 0.6 were mixed in a 1: 1 donor: recipient ratio, washed on LB medium, resuspended in 30 μL of LB and deposited on a 0.22 μm pore size cellulose acetate filters (Sartorius Stedim) in LB-agar plates previously incubated at 37ºC unless otherwise indicated. After 1 h, filters were removed with sterile tweezers and introduced in 1 mL LB, where cells were resuspended by vortexing for a few seconds. 1/10 serial dilutions were done and 10 μL drops were plated in LB agar plates with the appropriate selecting antibiotics for donors, recipients and transconjugants. Conjugation frequencies were obtained by dividing transconjugants per recipients (T/R). For conjugations in the presence of pUCP22-derived plasmids, isopropyl β-D-thiogalactoside (IPTG) was added to the conjugation mixture to a 0.1 mM IPTG final concentration. Means and standard deviations, as well as statistical tests, were calculated with GraphPad Prism1 (v 7.04) biostatistics software.

**Table 2:** Strains used in this study.

Strain	Phenotype	Reference
***Escherichia coli***		
DH5α	*F- end*A1 *gln*V44 *thi-1 rec*A1 *rel*A1 *gyr*A96 *deoR nupG*, *Φ80dlacZΔ*M15 *Δ(lacZYA-argF)*U169, *hsd*R17*(rK- mK+)*, *λ–*	[33]
BL21 (DE3)	F– *ompT* *gal* *dcm* *lon* *hsdSB*(*rB*–*mB*–) λ(DE3[*lacI* *lacUV5*-*T7p07* *ind1* *sam7* *nin5*]) [*malB*+]K-12(λS)	[34]
BL21(DE3)-SmR	Streptomycin resistant spontaneous mutant of BL21(DE3)	This work
C41 (DE3)	F- *ompT hsdSB (rB*- *mB*-*) gal dcm* (DE3)	[35]
β834(DE3)	F- *ompT hsdS*B(rB- mB-) *gal dcm met* (DE3)	[36]
TB10	TB10 is the result of a P1 transduction from DY329 into MG1655. It has a large amount of the λ prophage genome inserted into a biotin operon. The λ red genes α, β and γ are under the control of cI857, making it temperature inducible.	[37] and [38]
DY380	Sm^R^ λ Cl857 (cro^-^bioA) tet (DH10B)	[39]
BW27783	F-, Δ(*araD-araB*)567, ΔlacZ4787(::rrnB-3), λ-, *rph-1*, Δ(*rhaD-rhaB*)568, *hsd*R514 Δ(*araH-araF*)570(::FRT), Δ*araE*p-532::FRT, *φPcp8ar*aE535	[40]
BW27783-NxR	Nalidixic resistant spontaneous mutant of BW27783	[41]
BW27783-RifR	Rifampicin resistant spontaneous mutant of BW27783	[41]
MG1655	K-12 F–λ–*ilvG*–*rfb-50 rph-1*	[42]
MG1655-RifR	Rifampicin resistant spontaneous mutant of MG1655	This work
EcMR2Δ*mutS*	MG1655, *lacI- bla*, *bio-*, *lambda-Red1*, *mutS*–, cmR	[43]
***Pseudomonas putida***		
KT2440	Wild-type *P*. *putida* strain; mt-2 derivative cured of its plasmid (pWW0-)	[44]
EM178	KT2440 derivative; Δprophage1 Δprophage4 Δprophage3 Δprophage2	[45]
EM42	KT2440 derivative; Δprophage1 Δprophage4 Δprophage3 Δprophage2 Δ*tn7* Δ*endA-1* Δ*endA-2* Δ*hsdRMS* Δflagellum Δ*tn4652*	[45]
EM422	KT2440 derivative; Δ*hsdRMS*	From De Lorenzo group
KT2440 Δ*recA*	KT2440 derivative; Δ*recA*	From De Lorenzo group
KT2440 Δflagellum	KT2440 derivative; Δflagellum	From De Lorenzo group
KT2440 Δ*endA1*	KT2440 derivative; Δ*endA-1*	From De Lorenzo group
KT2440 Δ*endA2*	KT2440 derivative; Δ*endA-2*	From De Lorenzo group
KT2440 Δt*n7*	KT2440 derivative; Δ*tn7*	From De Lorenzo group
KT2440 Δ*tn4652* pSW	KT2440 derivative; Δ*tn4652 bearing pSW plasmid*	From De Lorenzo group
KT2440 Δ*tn4652*	KT2440 Δ*tn4652* derivative cured of pSW plasmid	This work
***Other bacteria ***		
*Salmonella* *typhimurium* LT2	*Salmonella enterica* subsp. *enterica* serovar *typhimurium* str. *LT2*	ATCC 700720
*Klebsiella* *pneumoniae* K6	*K*. *pneumoniae* subsp. *pneumoniae*. Clinical isolate from the Medical College of Virginia, 1994. Ap^R^ KmR, Cm^R^	ATCC 700603
*Acinetobacter* *baumannii*	Sm^R^ Ap^R^	ATCC 19606
*Vibrio cholerae* CIP106855	N16961 Rif^R^. Biovar Eltor, serovar O:1	CIP106855
*Agrobacterium tumefaciens* GMI9023	*Agrobacterium tumefaciens* C58 derivative cured of its plasmids (pTi-, pAT-)	[46]

**Table 3:** Plasmids used in this study.

Plasmid	Description	Phenotype	Size (Kb)	Reference
R388	R388 wild type plasmid	Su^R^ Tp^R^; (IncW)	33.9	[47]
pSU2007	R388 derivative; KnR cassette insertion	Su^R^ Tp^R^ Kn^R^; (IncW)	32.9	[48]
pET29c	Expression vector	Kn^R^; Rep (pMB1); Overexpression controlled by T7 promoter with a 6-HisTag.	5.4	Addgene
pUA66	GFP reporter plasmid	Kn^R^ pSC101 replicon	4.5	[49]
pUCP22	Shuttle Vector; Escherichia-Pseudomonas broad-host-range expression vector	Ap^R^ Gm^R^; Plac promoter.	4.7	[50]
pHERD20T	Shuttle Vector; Escherichia-Pseudomonas broad-host-range expression vector	Cb^R^; PBAD promoter and araC regulator.	5.1	[51]
pLGM21	pET29c::ardC	Kn^R^ T7 promoter	6.1	This work
pIC10	R388ΔkfrA-orf14	Tp^R^ Kn^R^	26.2	This work
pLGM25	R388ΔardC	Tp^R^ Kn^R^	33.9	This work
pLGM28	pET29c::ardC_E229A	Kn^R^ T7 promoter	6.1	This work
pLGM33	pSU2007 (ardC_E229A)	Tp^R^ Kn^R^	32.9	This work
pLGM36	pUCP22::ardC	Ap^R^ Gm^R^ Plac promoter	5.6	This work
pLGM37	pUCP22::ardC_E229A	Ap^R^ Gm^R^ Plac promoter	5.6	This work

### Transcriptomic analysis

Mixtures of *E*. *coli* BW27783-Nx^R^ (bearing pSU2007, pLGM25 or no plasmid) with *P*. *putida* KT2440 were carried out by the already described conjugation assay in a 5:1 donor: recipient ratio for 30 minutes. Harvested cells from the conjugation filter were treated with RNAprotect® Bacteria Reagent (Qiagen) and snap-frozen. Cells were lysed with lysozyme (Sigma) and proteinase K (Roche). Total RNA was extracted with RNeasy Mini Kit (Qiagen) and treated with RNase-free DNase (Qiagen) in column for DNA removal. Ambion TURBO DNA-free^™^ DNase Treatment was also applied for better DNA removal. RNA integrity and quality were validated by the Agilent RNA ScreenTape assay. The RNA integrity number equivalent (RINe) was assured to be above 8 to use the isolated RNA in the RNA-seq experiment.

Transcriptome libraries were prepared by Macrogen (Seoul, Korea) with Ribo-Zero rRNA Removal Kit and TruSeq Stranded mRNA sample preparation kit (Illumina). Libraries were sequenced by Macrogen on the Illumina HiSeq 4000 platform. The transcriptome libraries were paired-end sequenced with 100-bp reads. Raw reads in FASTQ format were quality analyzed with FastQC [52]. Reads were mapped against R388 (NCBI Accession number NC_028464.1), *E*. *coli* str. K-12 substr. MG1655 (U00096.3) and *P*. *putida* KT2440 (AE015451.2) sequences. The alignment of reads was done with Bowtie2 software [53]. Artemis program [54] was used to visualize the alignment and do the RPKM (reads per kilobase and million mapped reads) calculations. Genes with less than 10 RPKMs in all experimental conditions were removed from the analysis. DAVID online tool v6.8 [55] was used to test for gene ontology enrichment among the list of differentially expressed genes to do a functional classification.

### Protein expression and purification

Plasmids pLGM21 (pET29c::*ardC*) or pLGM28 (pET29c::*ardC_E229A*) were transformed into electrocompetent *Escherichia coli* BL21 (DE3) cells (Table 2). Transformed cells were grown in 1L LB medium, in the presence of kanamycin, at 37°C, with shaking, to an optical density of 0.5–0.6. The temperature was then reduced to 18°C and protein expression was induced with IPTG to a final concentration of 0.5 mM. Cells were allowed to grow for 16 h. The cultures were then centrifuged at 5,000 rpm and 4°C for 15 min and the resulting pellets were stored at −20°C. For protein purification, pellets were resuspended in buffer A (500 mM NaCl, 20 mM imidazole, 100 mM Tris-HCl pH 7.5) supplemented with protease inhibitor phenylmethylsulfonyl fluoride (PMSF) 1% (v/v). The slurry was sonicated in a Labsonic 2000 (B. Braun) equipment at 50% of potency for 3 cycles of 1.5 min at intervals of 1 min on ice. The lysed cells were then ultra-centrifuged at 100,000 g for 15 min at 4°C. Supernatants were loaded onto a 5 mL HisTrap HP column (GE Healthcare) previously equilibrated with buffer A. Proteins were eluted by an imidazole concentration gradient between buffer A and buffer B (300 mM NaCl, 500 mM imidazole, 100 mM Tris-HCl pH 7.5). ArdC containing fractions were pooled and diluted to a final NaCl concentration of 200 mM. The resulting protein was then loaded onto a 5 mL HiTrap Heparin HP (GE Healthcare) equilibrated with buffer C (100 mM Tris-HCl pH 7.5, 200 mM NaCl). Proteins were eluted by a linear gradient between buffer C and D (100 mM Tris-HCl pH 7.5, 1 M NaCl). ArdC containing fractions were concentrated using Amicon Ultra 30k Centrifugal filters (Millipore, Ireland) and loaded onto a Superdex 75 GL10_30 column (GE Healthcare) previously equilibrated with buffer E (100mM Tris-HCl pH 7.5, 1 mM EDTA, 300 mM NaCl).

To crystallize ArdC with a metal cofactor, cell lysis and the two first purification steps were done as described but with 1 mM MnCl_2_ in all buffers. Preparation of selenomethionine (SeMet)-labelled ArdC was also carried out as described above but using strain *E*. *coli* β834 (DE3) and minimal medium (SelenoMet Medium Base + SelenoMet Nutrient Mix) supplemented with SelenoMethionine Solution (Molecular Dimensions) as indicated by the manufacturer.

### Protein crystallization and structure determination

Crystals of ArdC and ArdC-SeMet were obtained using the sitting-drop vapor-diffusion method at 22°C by mixing 1.5 μL protein at 20 mg/mL concentration in 20 mM Tris-HCl, 50 mM NaCl, 1 mM EDTA buffer with an equal volume of the reservoir solution containing 0.1 M HEPES pH 7.5; 10% w/v polyethylene glycol 6,000 and 5% v/v (+/-)-2-methyl-2,4-pentanediol. 2-methyl-2 4-pentanediol (10–20% v/v) was added as cryoprotectant before diffraction experiments. ArdC-Mn crystallized at 12 mg/mL in 25% v/v ethylene glycol and crystals were cryoprotected with additional 15% glycerol.

For data collection, the crystals were flash-frozen in liquid nitrogen at 105 K. For single ArdC-SeMet crystals data was collected at 0.9793Å, the wavelength corresponding to the Selenium absorption maximum according to the fluorescence scan. Datasets were obtained at beamline XALOC at the ALBA Synchrotron Radiation Facility (Barcelona, Spain) with a Dectris PILATUS3 6M Pixel detector. Diffraction images were processed using iMosflm [56] and Scala [57] as part of the CCP4 package [58]. The structure was solved by single anomalous dispersion (SAD) phasing using the program AutoSol of the PHENIX package [59]. The refinement of the initial model was performed through several cycles by Phenix refine [59] until appropriate R factors were reached. Final manual modeling was done in COOT [60]. The ArdC-Mn structure was solved by MR using the ArdC structure as a template.

### Electrophoretic mobility shift assay (EMSA)

The binding ability of the ArdC protein to ssDNA, dsDNA, and dsDNA with ssDNA overhangs was tested by electrophoresis mobility shift assay (EMSA). 6FAM-labeled oligonucleotide Fluor-T87I2 (45 bases) was incubated alone or with T87I1 oligonucleotide (45 complementary bases), Mid1 oligonucleotide (13 5’ terminal complementary bases) or Mid2 oligonucleotide (27 3’ terminal complementary bases) (see S9 Table) in buffer containing 50mM Tris-HCl (pH 7.5) and 1mM EDTA for 5 min at 95°C and the mixtures were cooled down slowly to room temperature. 50 nM DNA was incubated with various concentrations of ArdC (0, 125 nM, 250 nM, 500 nM and 1 μM) in a reaction buffer [50 mM NaCl, 25 mM Tris-HCl (pH 7.5), 0.5 mM EDTA] at room temperature for 30 min. DNA-protein complexes were analyzed using non-denaturing polyacrylamide gel electrophoresis 10% (29:1) in cold Tris Borate EDTA (TBE) buffer 1x. Gels were run at 100 V for 75 min and analyzed using a Fujifilm fluorescent image analyzer Fla-5100. Experiments were repeated three times.

Extended Materials and Methods are included in S1 Text.

## Supporting information

S1 Text Supplementary Materials and Methods. (DOCX)

S1 Fig Genetic map of R388 plasmid. Genetic organization of the plasmid divided into functional modules and three different sectors: conjugation shadowed in orange, general maintenance in light blue and Ab^R^ and integration in grey. Region deleted in pLGM25 (shown in maroon) and pIC10 (shown in purple) are also shown. Adapted from [12].
(TIF)

S2 Fig Retardation and protection of ssDNA (M13mp18) by ArdC from degradation by HhaI. A) Agarose gel showing ssDNA retardation by ArdC under non-denaturing conditions. Lane 1: ssDNA (5.5 nM) in the presence of MgCl_2_. The vast majority of the molecules of M13mp18 ssDNA are circular (upper band), although some of them are present in the linear form (lower band). Lane 2: ssDNA and HhaI (7 U) in the presence of MgCl_2_. Lane 3–7: ssDNA and HhaI at increasing concentrations of ArdC (0.95 μM (3), 1.9 μM (4), 3.8 μM (5), 5.7 μM (6) and 7.8 μM (7)) in the presence of MgCl_2_. B) Agarose gel showing ssDNA protection by ArdC from HhaI digestion. Proteinase K and SDS were added to the samples before loading the gel to remove HhaI and ArdC proteins. Lane content as in A). C) SDS-PAGE gel showing the proteolytic activity of ArdC preincubated with ssDNA (M13mp18) to HhaI. Lane 1: ArdC (8 μM) in the presence of MgCl_2._ Lane 2: HhaI (70 U) in the presence of MgCl_2_. Lane 3: ArdC and HhaI in the presence of MgCl_2._ Lane 4–5: ArdC and HhaI in the presence of ssDNA (M13, 27.5 nM) with MgCl_2_ (4) or EDTA (5).
(TIF)

S3 Fig Mass spectrometry analysis for protein identification. A) Protein from a pull-down experiment cleaved from the gel and sent for mass spectrometry analysis is indicated by an arrow. B) MASCOT search result for the peptides obtained after digestion with trypsin (that cuts the C-term side of KR unless next residue is P) in SwissProt database for *P*. *putida* KT2440. Matched peptides with pp_0941 protein are shown in purple (sequence coverage of 45%). pp_0941 has 173 amino acids and a molecular weight of 20238 Da.
(TIF)

S4 Fig Differential expression of genes in experiment *ardC* ^*+*^ vs. *ardC—*to test the influence of ArdC in the three reference sequences. A) Differential expression of R388 genes. B) Differential expression of *E*. *coli* genes. C) Differential expression of *P*. *putida* genes. Upregulated genes with an RPKM fold change >2 are in the green zone, and downregulated genes with RPKM fold change <2 are in the orange zone.
(TIF)

S5 Fig Effect of ArdC on plasmid conjugative transfer from *E*. *coli* to *P*. *putida* wt and mutants at 30°C. The conjugation frequencies per recipient (T/R) into *P*. *putida* KT2440 WT strain or different mutants of *P*. *putida* KT2440 are shown. Conjugation was done for 1 h at 30°C. Horizontal bars represent the mean ± SD obtained for each dataset of N = 9 (t-test: ** p < 0.01).
(TIF)

S6 Fig Plasmids encoding TOPRIM and DUF1738 domains. Venn diagrams showing A) the proteins containing either DUF1738, TOPRIM or DUF1738+TOPRIM domains and B) plasmids encoding proteins containing DUF1738 and/or TOPRIM domains either in separate or multidomain proteins. Below, a panel indicates the fraction of plasmids potentially transmissible by conjugation (MOB^+^) for each condition. Proteins contained in RefSeq plasmid database version 90 were screened with the HMM profiles PF13362 for TOPRIM and PF08401 for DUF1378, using the hmmsearch function of HMMER 3.1b2 with the default parameters [61]. The presence of MOB relaxases was detected by using MOBscan [27]. Venn diagrams were built using the online tool http://bioinformatics.psb.ugent.be/webtools/Venn/.
(TIF)

S1 Table T_M_ value of ArdC in different solutions. (DOCX)

S2 Table Conjugation frequencies from *E*. *coli* to *P*. *putida*. (DOCX)

S3 Table TruSeq Stranded mRNA Illumina sequencing results and coverage for each condition. (DOCX)

S4 Table Percentage of reads aligned to the three reference sequences by Bowtie2. (DOCX)

S5 Table Distribution of the differentially upregulated or downregulated genes for the three reference sequences and conditions. (DOCX)

S6 Table Expression profile of R388 genes. (DOCX)

S7 Table Expression profile of upregulated *E*. *coli* genome-encoded genes in the absence of *ardC* in the plasmid. (DOCX)

S8 Table Expression profile of differentially upregulated *P*. *putida* genes in the presence/absence of *ardC*. (DOCX)

S9 Table Oligonucleotides used in this study. (DOCX)
