# Supplementary material for: ArdC, a ssDNA-binding protein with a metalloprotease domain, overpasses the recipient hsdRMS restriction system broadening conjugation host range
Source: PLoS Genet. 2020 Apr 29;16(4):e1008750. doi: 10.1371/journal.pgen.1008750 (PMC7213743; doi:10.1371/journal.pgen.1008750)
Supplement: S1 Text — (DOCX) [file pgen.1008750.s001.docx]

**Supplementary Materials and Methods**

Culture conditions and antibiotic usage

All bacterial strains were cultured in Luria-Bertani (LB) medium at 37 ºC except *Agrobacterium tumefaciens* *and Pseudomonas putida* for which the optimal growing temperature was 30 ºC. When required, the following antibiotics were used: 100 μg/mL ampicillin, 50 μg/mL kanamycin, 25 μg/mL chloramphenicol, 10 μg/mL gentamycin, 20 μg/mL nalidixic acid, 10 μg/mL tetracycline, 20 μg/mL trimethoprim, 50 μg/mL rifampicin, and 300 μg/mL streptomycin.

Construction of pET29c derived overexpression vectors

*ardC* coding sequence described by [1] was cloned into pET29c vector (Table 3) with a C-terminal 6xHis tag by Isothermal assembly [2]. *ardC* was obtained with “ArdC-Nterm” and “ArdC-Cterm” oligonucleotides and vector opened with “pET29CNdeI” and “pET29CXhoI” oligonucleotides (Table S9) by PCR with Phusion® DNA polymerase (Thermo Scientific). PCR products were digested with DpnI FD (Thermo Scientific) restriction enzyme and incubated in isothermal assembly reaction mixture [2] for 1 h at 50 °C.

This pET29c::*ardC* construction was mutated by Quick Change site directed mutagenesis method (Adapted from QuickChange II Site-Directed Mutagenesis Kit Protocol) to obtain pET29c::*ardC_E229A*. We used oligonucleotides *“*ArdC E229A d” and “ArdC E229A r” (Table S9) and Vent® DNA polymerase. PCR products were digested with DpnI FD (Thermo Scientific) restriction enzyme.

Construction of pUCP22 derived expression vectors

The *ardC* gene *(*NCBI Reference Sequence: NC_028464.1, coordinates 22591-23547) was amplified by PCR with oligonucleotides “ardC_rev(Hin)” and “ardC_fwd(Eco)” (Table S9) and Phusion® DNA polymerase (Thermo Scientific). The PCR product and the pUCP22 vector (Table 3) were digested with FastDigest EcoRI and HindIII (Thermo Scientific) restriction enzymes and ligated with T4 DNA ligase (Thermo Scientific).

The resulting pUCP22::*ardC* plasmid was subjected to Quick Change site-directed mutagenesis using PfuUltra II Hotstart PCR Master Mix (Agilent Technologies) and oligonucleotides “ArdC E229A d” and “ArdC E229A r” (Table S9) to generate pUCP22::*ardC_E229A.*

Construction of R388∆*kfrA-orf14* deletion mutant

R388Δ*kfrA-orf14* plasmid (pIC10) was constructed by a modification of the Wanner and Datsenko method [3]. “KfrAKamiK1” and “Orf14KamiK2” oligonucleotides (Table S9) were used to amplify by PCR with Phusion® DNA polymerase (Thermo Scientific) the Kn^R^ gene (with two added KpnI flanking sites) from the pUA66 vector (Table 3). The 973 bp DNA fragment was extracted from the gel, treated with FD DpnI restriction enzyme, and dialyzed against water. The strain used for recombination was *E. coli* DY380 (Table 2). R388 plasmid was introduced by conjugation to this strain and transconjugants (Sm^R^, Tp^R^) were grown at 30 °C o/n. The next day, a 1/50 dilution was done in LB and cells were grown until an OD_600_ of 0.5-0.7. Then, the culture was incubated at 42 °C for 20 min with shaking to induce the recombineering system. After this time, the cells were placed on ice for 20 min and made electrocompetent according to standard molecular biology protocols [4]. For transformation, 100 ng of the PCR product were used. Electroporation was performed at 2.5 keV and constant time (3-5 ms) in a Micropulser^TM^ electroporator (Bio-Rad). The cells were immediately recovered in 1 mL of sterile LB prewarmed at 30 °C, and let at this temperaturefor 2 h. Finally, the cells were plated in LB agar plates supplemented with Kn and incubated at 30 °C o/n. Colony checking PCRs were done with Taq polymerase to verify the substitution. Plasmids from some positive colonies were conjugated to DH5α cells by matings for 1 h at 30 ºC and selection on Kn Nx LB agar plates. Another checking colony PCR was carried out to confirm that we had the mutant plasmid isolated.

Construction of R388∆*ardC* deletion mutant

R388Δ*ardC* plasmid (pLGM25) was constructed by a modification of the Wanner and Datsenko method [3]. “N_Kn_promoter_Wanner” and “C_Kn_Wanner” oligonucleotides (Table S9) were used to amplify by PCR with Vent® polymerase the Kn^R^ cassette with its promoter from pET29c vector (Table 3). The 1227 bp DNA fragment was extracted from the gel, treated with FD DpnI restriction enzyme, and dialyzed against water. The strain used for recombination was *E. coli* TB10 (Table 2). R388 plasmid was introduced by conjugation to this strain and transconjugants (Tc^R^ and Tp^R^) were grown at 30 °C o/n. The next day, a 1/70 dilution was done in LB and cells were grown until an OD_600_ of 0.5. Then, the culture was incubated at 42 °C for 15 min with shaking to induce the recombineering system. After this time, the cells were made electrocompetent according to standard molecular biology protocols [4]. For transformation, 100 ng of the PCR product were used. Electroporation was performed at 2.5 keV and time constant (3-5 ms) in a Micropulser^TM^ electroporator (Bio-Rad), cells were immediately recovered in 1 mL of sterile LB prewarmed at 30 °C. Then, cells were let at 30 °C for 3 h. Then, the cells were plated in Tc and Kn at half the normal antibiotic concentration. After one day, colonies were restricken in a plate with the normal antibiotic concentration. Colony checking PCRs were done with Taq polymerase with oligonucleotides “Up”, “Down”, “Middle_Up” and “Middle_Down” (Table S9) to verify the substitution. Plasmid DNA from some of the positive colonies was extracted and introduced in DH5α cells by electroporation to make sure that only one type of plasmid (mutated or WT) entered each cell. Another checking colony PCR was done for the selected mutated colonies with oligonucleotides “ArdC-Cterm” and “ArdC-Nterm” (Table S9) to make sure that they do not amplify any fragment and thus, confirm that we had the mutant plasmid isolated.

Mass spectrometry analysis for protein identification

To identify the putative ArdC protease target obtained by the pull-down assay, a protein identification assay was done by Liquid chromatography-tandem mass spectrometry (LC-MS/MS). The selected protein band was subjected to in-gel tryptic digestion according to [5], with minor modifications. Gel pieces were swollen in digestion buffer containing 50 mM NH_4_HCO_3_ and 12.5 ng/μL proteomics grade trypsin (Roche, Basel, Switzerland), and the digestion processed at 37 °C o/n. The supernatant was recovered and peptides were extracted twice: first, with 25 mM NH_4_HCO_3_ and acetonitrile (ACN), and then with 0.1% (v/v) trifluoroacetic acid and ACN. The recovered supernatants and extracted peptides were pooled, dried in a SpeedVac (ThermoElectron, Waltham, MA), dissolved in 10 μL of 0.1 % (v/v) formic acid (FA), and sonicated for 5 min. LC-MS/MS spectra were obtained using a SYNAPT HDMS mass spectrometer (Waters, Milford, MA) interfaced with a nanoAcquity UPLC System (Waters). An aliquot (8 μL) of each sample was loaded onto a Symmetry 300 C18, 180 μm x 20 mm precolumn (Waters) and washed with 0.1 % (v/v) FA for 3 min at a flow rate of 5 μL/min. The precolumn was connected to a BEH130 C18, 75 μm × 200 mm, 1.7 μm (Waters), equilibrated in 3 % (v/v) ACN and 0.1 % (v/v) FA. Peptides were eluted with a 30 min linear gradient of 3−60 % (v/v) ACN directly onto a homemade nano-electrospray capillary tip. The capillary voltage was set to 3,500 V and data-dependent MS/ MS acquisitions performed on precursors with charge states of 2, 3, or 4 over a survey m/z range of 350−1990. Raw files were processed with VEMS [6] and searched against the NCBI non-redundant (nr) database restricted to Proteobacteria (version 20171205, 49911253 sequences) using the online MASCOT server (Matrix Science Ltd., London; http://www.matrixscience.com). Protein identification was carried out by adopting the carbamidomethylation of Cys as fixed modification and the oxidation of Met as variable modification. Up to one missed cleavage site was allowed, and values of 50 ppm and 0.1 Da were set for peptide and fragment mass tolerances, respectively. Mass spectrometry analysis was performed in the Proteomics Core Facility-SGIKER (a member of ProteoRed-ISCIII) at the University of the Basque Country, UPV/EHU.

DNA-binding and protection assays

The assay was done under non-denaturing conditions to see DNA binding and retardation in parallel to under denaturing and proteolytic conditions: M13mp18 ssDNA (7.2 Kb, 5.5 nM final concentration) was incubated with increasing concentrations of ArdC for 10 min at RT in a total volume of 20 µL binding buffer: 10 mM Tris-HCl, 10 mM NaCl and 10 mM MgCl_2_. Then, 7 U of HhaI were added and incubated for 20 min at 37 °C. Afterward, 2.5 µL of DNA loading buffer were added to 10 µL of the sample. Samples were subjected to electrophoresis on a 1 % agarose gel with SYBER safe for 30 min at 120 V. 1.5 µL of proteinase K at 20 mg/mL and 1 µL SDS 10 % were added to the remaining 10 µL of the sample and the mixture was incubated for another 20 min at 37 °C. Reactions were mixed with 2.5 µL DNA loading buffer and electrophoresed in a 1 % agarose gel with SYBER safe for 30 min at 120V.

Proteolytic activity assay

ArdC proteolytic activity was analyzed using a modification of the method described by [7] for the study of IrrE metalloprotease. ArdC at a final concentration of 8 µM in 20 µL was incubated in buffer P (15 mM Tris-HCl pH 7.5, 15 mM NaCl and 15 mM of MgCl_2_ or 15 mM EDTA) and 27.5 nM M13 ssDNA for 10 min at RT. Then 70 U of HhaI were added and incubated for 20 min at 37 °C. Reactions were stopped by the addition of 20 µL of protein loading buffer 2x (400 mM Tris-HCl pH 6.8, 4 % SDS, 30 % glycerol and 0.04 % bromophenol blue) and boiled for 5 min. A 12 % SDS-PAGE was performed for 60 min at 180 V.

Site-directed MAGE *in vivo* mutagenesis method

To perform point mutation in pSU2007 and construct pLGM33, we used the non-automated version of the MAGE (Multiplex Automated Genome Engineering) method described by [8]. “ArdC_E229A_MAGE” (Table S9), a 90 base oligonucleotide containing the mutation in the middle and two phosphorothioate (PS) bonds in the 5’ end was designed. The phosphorothioate bond substitutes a sulfur atom for a non-bridging oxygen in the phosphate backbone of the primer. This modification in the internucleotide bond makes the primer resistant to exonuclease degradation. EcMR2Δ*mutS E. coli* strain (Table 2) was used. These cells were cultured at 30 °C when recombination was not needed. pSU2007 plasmid was introduced by conjugation into this strain and transconjugants (Kn^R^ and Rif^R^) were grown at 30 °C o/n. The next day, a 1/40 dilution was done in LB and cells were grown until an OD_600_ of 0.5. Then culture was incubated at 42 °C for 15 min with shaking to induce the recombineering system and after this time cells were made electrocompetent according to standard molecular biology protocols [4] except for the last wash, when cells were resuspended in 50 µL of a 1 µM oligonucleotide suspension so the mixture is ready for electroporation. Once recovered in 1 mL LB, cultures were grown at 30 °C. After 2 h, 50 µL were plated in Kn Rif plates and the rest of the volume was grown o/n labeled as cycle #1 until the next day when the protocol was repeated. When needed, stocks were saved at -80 °C in 25 % glycerol for further analysis. After 10 cycles, plasmid extraction from some colonies was done and the PCR product obtained with Phusion® polymerase and oligonucleotides “Up” and “Down”, was sent to sequence with “Down” oligonucleotide. The colony that gave an overlapping pick at the sequencing panel in the mutagenic position was further analyzed. Its plasmid DNA was extracted and electroporated in DH5α, and PCR fragments obtained with primers “Up” and “Down” were amplified from some of the colonies and sequenced. The process was repeated until a clean mutant pick was obtained for the desired position.

Thermal stability assay based on fluorescence

20 µL samples containing ArdC at 2.5 µM in 100 mM Tris-HCl pH 7.5 and 500 mM NaCl buffer containing 1mM of EDTA or 1mM of the metal to be analyzed and the SYPRO® Orange (Invitrogen) non-polar dye at a 2x final concentration were evaluated in a StepOnePlus^TM^ Real-Time PCR System (Thermo Fisher). For measuring SYPRO® Orange (excitation: 470 nm/ Emission: 570 nm), filter for NED™ dye was used, with excitation at 546 nm and emission of 575 nm. The temperature was raised from 25 °C to 85 °C at 0.5 °C per minute, measuring the fluorescence every 0.5 °C. T_M_ was determined as the maximum of the fluorescence versus temperature variation (dF/dT). The experiments were done by duplicate.

References

1. Belogurov AA, Delver EP, Agafonova O V, Belogurova NG, Lee LY, Kado CI. Antirestriction protein Ard (Type C) encoded by IncW plasmid pSa has a high similarity to the “protein transport” domain of TraC1 primase of promiscuous plasmid RP4. J Mol Biol. 2000;296: 969–977. doi:10.1006/jmbi.1999.3493

2. Gibson DG, Young L, Chuang RY, Venter JC, Hutchison CA, Smith HO. Enzymatic assembly of DNA molecules up to several hundred kilobases. Nat Methods. 2009;6: 343–345. doi:10.1038/nmeth.1318

3. Datsenko KA, Wanner BL. One-step inactivation of chromosomal genes in Escherichia coli K-12 using PCR products. Proc Natl Acad Sci. 2000;97: 6640–6645. doi:10.1073/pnas.120163297

4. Sambrook J, Russell DW. Molecular Cloning: A Laboratory Manual. 3rd ed. Molecular Cloning: a laboratory manual. Cold Spring Harbor, NY: Cold Spring Harbor Laboratory Press; 2001.

5. Shevchenko A, Wilm M, Vorm O, Mann M. Mass spectrometric sequencing of proteins from silver-stained polyacrylamide gels. Anal Chem. 1996;68: 850–858. doi:10.1021/ac950914h

6. Matthiesen R, Trelle MB, Højrup P, Bunkenborg J, Jensen ON. VEMS 3.0: Algorithms and computational tools for tandem mass spectrometry based identification of post-translational modifications in proteins. J Proteome Res. 2005;4: 2338–2347. doi:10.1021/pr050264q

7. Ludanyi M, Blanchard L, Dulermo R, Brandelet G, Bellanger L, Pignol D, et al. Radiation response in Deinococcus deserti: IrrE is a metalloprotease that cleaves repressor protein DdrO. Mol Microbiol. 2014;94: 434–449. doi:10.1111/mmi.12774

8. Wang HH, Isaacs FJ, Carr PA, Sun ZZ, Xu G, Forest CR, et al. Programming cells by multiplex genome engineering and accelerated evolution. Nature. 2009;460: 894–898. doi:10.1038/nature08187
