# Supplementary material for: ArdC, a ssDNA-binding protein with a metalloprotease domain, overpasses the recipient hsdRMS restriction system broadening conjugation host range
Source: PLoS Genet. 2020 Apr 29;16(4):e1008750. doi: 10.1371/journal.pgen.1008750 (PMC7213743; doi:10.1371/journal.pgen.1008750)
Supplement: S1 Table — (DOCX) [file pgen.1008750.s008.docx]

S1 Table. T_M_ value of ArdC in different solutions.

| Condition | **T_m_ (°C) ^a^** |
| --- | --- |
| - | 56 |
| 1 mM EDTA | 55 |
| 1 mM NiCl2 | 62 |
| 1 mM CaCl2 | 55 |
| 1 mM MgCl2 | 55 |
| 1 mM MnCl2 | 60 |
| 1 mM ZnCl2 | 56 |
| 1 mM CuCl2 | 59 |
| 1 mM CoCl2 | 69 |
| 1 mM FeCl3 | 55 |

^a^ T_M_ values are for protein in 100 mM Tris-HCl pH 7.5, 500 mM NaCl alone or plus 1 mM EDTA or 1 mM metals. T_M_ was calculated by fluorescence measurements using SYPRO® Orange dye.
