# Supplementary material for: ArdC, a ssDNA-binding protein with a metalloprotease domain, overpasses the recipient hsdRMS restriction system broadening conjugation host range
Source: PLoS Genet. 2020 Apr 29;16(4):e1008750. doi: 10.1371/journal.pgen.1008750 (PMC7213743; doi:10.1371/journal.pgen.1008750)
Supplement: S2 Table — (DOCX) [file pgen.1008750.s009.docx]

S2 Table. Conjugation frequencies from *E. coli* to *P. putida* ^a^*.*

| **Condition** | **Donor strain** | **Donors ^b^ (D)** | **Recipients ^c^ (R)** | **Transconjugants ^d^ (T)** | **D : R** | **Freq.**  **(T/D)** | **Freq. (T/R)** |
| --- | --- | --- | --- | --- | --- | --- | --- |
| **NP** | BW27783 | 1.3E+06 | 2.0E+05 | 0.0E+00 | 6.5 | <1.0E-08 | <1.0E-08 |
| ***ardC +*** | BW27783 + pSU2007 | 1.4E+06 | 2.5E+05 | 2.9E+04 | 5.6 | 2.1E-02 | 1.2E-01 |
| ***ardC-*** | BW27783 + pLGM25 | 1.2E+06 | 4.0E+05 | 1.0E+01 | 3.0 | 8.3E-06 | 2.5E-05 |

a Conjugations were performed from *E. coli* BW27783-Nx^R^ containing no plasmid (NP), pSU2007 or pLGM25 as donor cells to *P. putida* KT2440 as recipient cells in all cases for 30 min at 37 °C. The conjugation frequencies per donor (Freq. T/D) and per recipient (Freq. T/R) are shown. b Donors were selected in LB agar plates containing Kn Nx. c Recipients were selected in LB agar plates containing Ap Cm. d Transconjugants were selected in LB agar plates containing Kn Cm.
