# Supplementary material for: ArdC, a ssDNA-binding protein with a metalloprotease domain, overpasses the recipient hsdRMS restriction system broadening conjugation host range
Source: PLoS Genet. 2020 Apr 29;16(4):e1008750. doi: 10.1371/journal.pgen.1008750 (PMC7213743; doi:10.1371/journal.pgen.1008750)
Supplement: S3 Table — (DOCX) [file pgen.1008750.s010.docx]

S3 Table. TruSeq® Stranded mRNA Illumina sequencing results and coverage for each condition.

| **Sample ID** | **Total read bases (bp)** | **Total reads ^a^ (N)** | **GC (%)** | **AT (%)** | **Q20 (%) ^b^** | **Q30 (%) ^c^** | **Coverage ^d^** |
| --- | --- | --- | --- | --- | --- | --- | --- |
| **NP** | 22,467,804,106 | 222,453,506 | 54.59 | 45.41 | 97.96 | 94.54 | 2055x |
| ***ardC +*** | 19,110,456,642 | 189,212,442 | 54.55 | 45.45 | 97.95 | 94.45 | 1743x |
| ***ardC -*** | 23,255,871,352 | 230,256,152 | 56.05 | 43.95 | 97.9 | 94.33 | 2121x |

^a^ Total reads refers to the sum of reads 1 and reads 2 from strand-specific sequencing. ^b^ Q20 stands for phred quality score over 20. ^c^ Q30 stands for phred quality score over 30. ^d^ Coverage was calculated according to the formula C=L·N/G from the length of reads (L = 100), N and the genome size (G) being the sum of all genomes present in the sample.
