# Supplementary material for: ArdC, a ssDNA-binding protein with a metalloprotease domain, overpasses the recipient hsdRMS restriction system broadening conjugation host range
Source: PLoS Genet. 2020 Apr 29;16(4):e1008750. doi: 10.1371/journal.pgen.1008750 (PMC7213743; doi:10.1371/journal.pgen.1008750)
Supplement: S4 Table — (DOCX) [file pgen.1008750.s011.docx]

**S4 Table. Percentage of reads aligned to the three reference sequences by Bowtie2.**

| **Sequenced sample** | **total reads** | **% mapped to *E. coli*** | **% mapped to *P. putida*** | **% mapped to R388** |
| --- | --- | --- | --- | --- |
| **NP_1** | 111,226,753 | 52.81 | 46.70 | 0.00 |
| **NP_2** | 111,226,753 | 52.74 | 45.92 | 0.00 |
| ***ardC +*_1** | 94,606,221 | 51.84 | 43.72 | 1.73 |
| ***ardC +*_2** | 94,606,221 | 51.43 | 43.18 | 1.84 |
| ***ardC -*_1** | 115,128,076 | 35.35 | 62.63 | 1.03 |
| ***ardC -*_2** | 115,128,076 | 35.40 | 61.76 | 1.03 |
