# Supplementary material for: ArdC, a ssDNA-binding protein with a metalloprotease domain, overpasses the recipient hsdRMS restriction system broadening conjugation host range
Source: PLoS Genet. 2020 Apr 29;16(4):e1008750. doi: 10.1371/journal.pgen.1008750 (PMC7213743; doi:10.1371/journal.pgen.1008750)
Supplement: S5 Table — (DOCX) [file pgen.1008750.s012.docx]

S5 Table. Distribution of the differentially upregulated or downregulated genes for the three reference sequences ^and^ conditions ^a^.

|  | **R388** | ***E. coli*** | ***P. putida*** |
| --- | --- | --- | --- |
| ***ardC +/ardC -*** | ↑19 (40.43 %)  ↓1 (2.13 %) | ↑515 (11.45 %)  ↓14 (0.31 %) | ↑76 (1.31 %)  ↓20 (0.35 %) |
| ***ardC +/* NP** | - | ↑29 (0.64 %)  ↓52 (1.16 %) | ↑59 (1.02 %)  ↓75 (1.30 %) |
| ***ardC -/* NP** | - | ↑60 (1.33 %)  ↓599 (13.32 %) | ↑25 (0.43 %)  ↓67 (1.16 %) |

^a^ Upregulated genes (↑): RPKM fold change ≥ 2 for each pair of experiments compared and downregulated genes (↓) RPKM fold change ≤ 2. Percentage respect total genes for each reference sequence is also shown.
