# Supplementary material for: ArdC, a ssDNA-binding protein with a metalloprotease domain, overpasses the recipient hsdRMS restriction system broadening conjugation host range
Source: PLoS Genet. 2020 Apr 29;16(4):e1008750. doi: 10.1371/journal.pgen.1008750 (PMC7213743; doi:10.1371/journal.pgen.1008750)
Supplement: S6 Table — (DOCX) [file pgen.1008750.s013.docx]

S6 Table. Expression profile of R388 genes.

|  | **RPKMs** | | **Fold Change** | **Information** | |
| --- | --- | --- | --- | --- | --- |
| **Locus tag** | ***ardC^+^*** | ***ardC ^-^*** | ***ardC ^+^*/**  ***ardC ^-^* *^a^*** | **Gene name** | **Protein function** |
| R388_0003 | 23018.35 | 984.59 | 23.38 | *trwN* | Lysozyme-like domain |
| R388_0002 | 24092.60 | 1159.94 | 20.77 | *kikA* | Causes reversible growth inhibition |
| R388_0022 | 39521.88 | 2673.90 | 14.78 | *orf14* | Putative cold shock DNA-binding domain-like |
| R388_0024 | 40585.67 | 2781.78 | 14.59 | *orf12* | Putative Type I R-M system methyltransferase subunit |
| R388_0023 | 33624.88 | 2762.11 | 12.17 | *klcB* | Putative transcriptional regulator |
| R388_0046 | 238132.80 | 30898.27 | 7.71 | *orf45* |  |
| R388_0047 | 77020.56 | 10164.97 | 7.58 | *orf46* |  |
| R388_0004 | 86510.91 | 13805.67 | 6.27 | *korA* | Putative transcriptional repressor |
| R388_0005 | 87087.27 | 16717.39 | 5.21 | *trwL* | TrbC/VirB2 family |
| R388_0006 | 61668.86 | 16422.23 | 3.76 | *trwM* | Type IV secretory pathway, VirB3-like protein |
| R388_0012 | 18435.34 | 5436.83 | 3.39 | *trwG* | VirB8-like protein |
| R388_0018 | 8152.53 | 2603.73 | 3.13 | *trwA* | DNA binding/nic-cleavage accessory protein |
| R388_0011 | 28950.89 | 10600.22 | 2.73 | *trwH* | Prokaryotic membrane lipoprotein lipid attachment site |
| R388_0028 | 15084.93 | 6244.13 | 2.42 | *ssb* | Single stranded DNA binding protein |
| R388_0007 | 22439.94 | 9952.14 | 2.25 | *trwK* | Type IV secretion/conjugal transfer ATPase, VirB4 family |
| R388_0013 | 9409.23 | 4177.71 | 2.25 | *trwF* | VirB9/CagX/TrbG, a component of the type IV secretion system |
| R388_0027 | 65575.23 | 29438.02 | 2.23 | *ardK* | Putative transcriptional repressor |
| R388_0029 | 34339.57 | 15685.98 | 2.19 | *orf9* |  |
| R388_0026 | 103954.11 | 55001.41 | 1.89 | *Ldr2* | Long direct repeat |
| R388_0017 | 9785.44 | 5591.23 | 1.75 | *trwB* | Type IV secretion-system coupling protein DNA-binding domain |
| R388_0020 | 86196.49 | 50700.52 | 1.70 | *stbB* | Protein involved in plasmid partition |
| R388_0019 | 60845.67 | 35815.90 | 1.70 | *stbA* | Protein involved in plasmid partition |
| R388_0009 | 18993.55 | 11419.67 | 1.66 | *eex* | Entry exclusion |
| R388_0008 | 17432.89 | 10809.77 | 1.61 | *trwJ* | VirB5 protein family |
| R388_0014 | 5650.80 | 3632.44 | 1.56 | *trwE* | Type IV secretion system protein VirB10 |
| R388_0015 | 5830.68 | 3961.39 | 1.47 | *trwD* | ATP hydrolase. VirB11-like protein |
| R388_0040 | 34281.28 | 24217.12 | 1.42 | *intI1* | Integron integrase/recombinase |
| R388_0001 | 8003.05 | 5809.62 | 1.38 | *korB* | Domain in histone-like proteins of HNS family |
| R388_0021 | 62283.80 | 46346.24 | 1.34 | *stbC* | Putative ribonucleotide reductase-like. Ferritin-like domain |
| R388_0016 | 8207.78 | 6410.90 | 1.28 | *trwC* | DNA helicase/Relaxase |
| R388_0010 | 7092.39 | 5575.50 | 1.27 | *trwI* | TrbL/VirB6 plasmid conjugative transfer protein |
| R388_0041 | 1365552.50 | 1118653.88 | 1.22 | *dhfr* | Dihydrofolate reductase. Confers resistance to trimethoprim |
| R388_0032 | 15405.67 | 13371.10 | 1.15 | *Ldr1* | Long direct repeat |
| R388_0025 | 40748.49 | 37204.41 | 1.10 | *Ldr2* | Long direct repeat |
| R388_0039 | 1390.29 | 1303.33 | 1.07 | *tnpM* | Truncated transposase |
| R388_0036 | 10110.16 | 10802.69 | 0.94 | *kfrA* | Plasmid replication region DNA-binding N-term; Region: KfrA_N |
| R388_0038 | 7957.77 | 8557.84 | 0.93 | *resP* | Putative resolvase |
| R388_0034 | 7687.23 | 8633.32 | 0.89 | *nuc2* | Putative DNAse |
| R388_0035 | 7246.52 | 8560.23 | 0.85 | *nuc1* | DNAse |
| R388_0030 | 13688.44 | 16890.83 | 0.81 | *orf7-8* |  |
| R388_0037 | 6113.80 | 7568.30 | 0.81 | *repA* | DNA replication initiation protein |
| R388_0033 | 4695.06 | 5957.16 | 0.79 | *osa* | Fertility inhibition factors, including OSA and FiwA, related to the ParB/Srx superfamily |
| R388_0045 | 32660.89 | 44679.79 | 0.73 | *orf5* | Acetyltransferase |
| R388_0042 | 129422.65 | 531165.69 | 0.24 | *orfA* | Putative protein-glutamine gamma-glutamyltransferase |

^a^ List is ordered from highest to lowest according to the Fold Change (ardC ^+^/ardC^-^) column.
