# Supplementary material for: ArdC, a ssDNA-binding protein with a metalloprotease domain, overpasses the recipient hsdRMS restriction system broadening conjugation host range
Source: PLoS Genet. 2020 Apr 29;16(4):e1008750. doi: 10.1371/journal.pgen.1008750 (PMC7213743; doi:10.1371/journal.pgen.1008750)
Supplement: S7 Table — (DOCX) [file pgen.1008750.s014.docx]

S7 Table. Expression profile of upregulated *E. coli* genome-encoded genes in the absence of *ardC* in the plasmid ^a^.

|  | **RPKMs** | | | **Fold Change** | | **Information** | |
| --- | --- | --- | --- | --- | --- | --- | --- |
| **Locus**  **tag** | **NP** | ***ardC^+^*** | ***ardC^-^*** | ***ardC ^-^***  **/NP** | ***ardC^-^ / ardC ^+ a^*** | **Gene name** | **Protein function** |
| b1389 | 10.05 | 18.70 | 1.78 | 0.18 | 0.10 | *paaB* | putative ring 1 |
| b1388 | 6.68 | 13.43 | 1.37 | 0.20 | 0.10 | *paaA* | ring 1 |
| b1391 | 8.70 | 13.41 | 1.47 | 0.17 | 0.11 | *paaD* | ring 1 |
| b1390 | 9.26 | 13.93 | 1.73 | 0.19 | 0.12 | *paaC* | ring 1 |
| b4618 | 1627.26 | 2354.32 | 298.09 | 0.18 | 0.13 | *tisB* | toxic membrane persister formation peptide |
| b2699 | 1014.00 | 1524.69 | 220.59 | 0.22 | 0.14 | *recA* | DNA recombination and repair protein |
| b3117 | 133.29 | 96.77 | 15.08 | 0.11 | 0.16 | *tdcB* | L-threonine dehydratase |
| b3113 | 135.27 | 92.33 | 15.29 | 0.11 | 0.17 | *tdcF* | putative reactive intermediate deaminase |
| b1882 | 10.98 | 13.81 | 2.51 | 0.23 | 0.18 | *cheY* | chemotaxis regulator transmitting signal to flagellar motor component |
| b3114 | 130.11 | 91.85 | 18.50 | 0.14 | 0.20 | *tdcE* | pyruvate formate-lyase 4/2-ketobutyrate formate-lyase |
| b2616 | 124.90 | 170.53 | 34.89 | 0.28 | 0.20 | *recN* | recombination and repair protein |
| b3116 | 173.47 | 124.93 | 26.60 | 0.15 | 0.21 | *tdcC* | L-threonine/L-serine transporter |
| b1848 | 729.62 | 1007.70 | 222.17 | 0.30 | 0.22 | *yebG* | DNA damage-inducible protein regulated by LexA |
| b2457 | 9.15 | 12.32 | 2.74 | 0.30 | 0.22 | *eutM* | ethanolamine utilization protein |
| b4471 | 76.28 | 48.09 | 10.83 | 0.14 | 0.23 | *tdcG* | L-serine dehydratase 3 |
| b3115 | 160.32 | 114.45 | 26.31 | 0.16 | 0.23 | *tdcD* | propionate kinase/acetate kinase C |
| b1184 | 12.76 | 23.58 | 5.49 | 0.43 | 0.23 | *umuC* | translesion error-prone DNA polymerase V subunit |
| b2698 | 98.98 | 108.24 | 25.65 | 0.26 | 0.24 | *recX* | regulatory protein for RecA |
| b1847 | 607.63 | 745.03 | 176.58 | 0.29 | 0.24 | *yebF* | extracellular Colicin M immunity family protein |
| b4606 | 115.33 | 96.49 | 23.21 | 0.20 | 0.24 | *ypfM* | stress-induced small enterobacterial protein |
| b0958 | 405.53 | 502.27 | 121.69 | 0.30 | 0.24 | *sulA* | SOS cell division inhibitor |
| b4408 | 799.52 | 1238.41 | 305.51 | 0.38 | 0.25 | *csrB* | CsrA-binding sRNA |
| b1846 | 162.91 | 224.22 | 57.75 | 0.35 | 0.26 | *yebE* | DUF533 family inner membrane protein |
| b4062 | 98.23 | 142.63 | 39.14 | 0.40 | 0.27 | *soxS* | superoxide response regulon transcriptional activator |
| b1183 | 24.29 | 32.87 | 9.08 | 0.37 | 0.28 | *umuD* | translesion error-prone DNA polymerase V subunit |
| b3157 | 67.94 | 63.89 | 18.85 | 0.28 | 0.30 | *yhbT* | SCP-2 sterol transfer family protein |
| b1396 | 12.64 | 13.11 | 3.93 | 0.31 | 0.30 | *paaI* | hydroxyphenylacetyl-CoA thioesterase |
| b2977 | 71.52 | 109.65 | 34.03 | 0.48 | 0.31 | *glcG* | DUF336 family protein |
| b2979 | 42.25 | 60.01 | 18.83 | 0.45 | 0.31 | *glcD* | glycolate oxidase subunit |
| b3118 | 27.24 | 23.93 | 7.60 | 0.28 | 0.32 | *tdcA* | tdc operon transcriptional activator |
| b1415 | 137.13 | 153.19 | 48.87 | 0.36 | 0.32 | *aldA* | aldehyde dehydrogenase A |
| b3565 | 7.74 | 10.25 | 3.29 | 0.42 | 0.32 | *xylA* | D-xylose isomerase |
| b1283 | 129.02 | 194.74 | 62.81 | 0.49 | 0.32 | *osmB* | osmotically and stress inducible lipoprotein |
| b0896 | 15.67 | 12.86 | 4.16 | 0.27 | 0.32 | *dmsC* | dimethyl sulfoxide reductase |
| b2203 | 31.13 | 48.49 | 15.80 | 0.51 | 0.33 | *napB* | nitrate reductase |
| b2976 | 58.78 | 73.60 | 24.03 | 0.41 | 0.33 | *glcB* | malate synthase G |
| b1881 | 7.77 | 10.20 | 3.41 | 0.44 | 0.33 | *cheZ* | chemotaxis regulator |
| b3744 | 89.16 | 58.36 | 19.81 | 0.22 | 0.34 | *asnA* | asparagine synthetase A |
| b0162 | 20.05 | 20.52 | 7.01 | 0.35 | 0.34 | *cdaR* | carbohydrate diacid regulon transcriptional regulator |
| b2666 | 30.11 | 45.32 | 15.52 | 0.52 | 0.34 | *yqaE* | cyaR sRNA-regulated protein |
| b4468 | 30.29 | 41.00 | 14.11 | 0.47 | 0.34 | *glcE* | glycolate oxidase FAD binding subunit |
| b2009 | 30.02 | 29.51 | 10.21 | 0.34 | 0.35 | *sbmC* | DNA gyrase inhibitor |
| b3156 | 59.25 | 52.49 | 18.52 | 0.31 | 0.35 | *yhbS* | GNAT family putative N-acetyltransferase |
| b1256 | 11.31 | 12.13 | 4.28 | 0.38 | 0.35 | *ompW* | outer membrane protein W |
| b2665 | 20.15 | 34.86 | 12.49 | 0.62 | 0.36 | *ygaU* | uncharacterized protein |
| b0643 | 10.47 | 10.60 | 3.90 | 0.37 | 0.37 | *ybeL* | DUF1451 family protein |
| b4487 | 27.84 | 30.61 | 11.70 | 0.42 | 0.38 | *yjdP* | putative periplasmic protein |
| b0627 | 210.33 | 238.14 | 91.15 | 0.43 | 0.38 | *tatE* | TatABCE protein translocation system subunit |
| b2204 | 30.88 | 36.76 | 14.08 | 0.46 | 0.38 | *napH* | ferredoxin-type protein |
| b0019 | 84.61 | 106.53 | 41.08 | 0.49 | 0.39 | *nhaA* | sodium-proton antiporter |
| b2206 | 31.27 | 40.52 | 15.63 | 0.50 | 0.39 | *napA* | nitrate reductase |
| b1518 | 10.18 | 10.23 | 3.95 | 0.39 | 0.39 | *lsrG* | autoinducer-2 (AI-2) degrading protein LsrG |
| b2202 | 43.74 | 54.95 | 21.23 | 0.49 | 0.39 | *napC* | quinol dehydrogenase |
| b3105 | 39.33 | 44.25 | 17.11 | 0.43 | 0.39 | *yhaJ* | LysR family putative transcriptional regulator |
| b1744 | 9.99 | 11.41 | 4.41 | 0.44 | 0.39 | *astE* | succinylglutamate desuccinylase |
| b4467 | 36.49 | 42.80 | 16.61 | 0.46 | 0.39 | *glcF* | glycolate oxidase 4Fe-4S iron-sulfur cluster subunit |
| b4090 | 17.52 | 19.21 | 7.49 | 0.43 | 0.39 | *rpiB* | ribose 5-phosphate isomerase B/allose 6-phosphate isomerase |
| b4518 | 20.71 | 25.01 | 9.83 | 0.47 | 0.39 | *ymdF* | KGG family protein |
| b2205 | 31.24 | 40.51 | 16.01 | 0.51 | 0.40 | *napG* | ferredoxin-type protein |
| b0710 | 18.42 | 26.99 | 10.67 | 0.58 | 0.40 | *ybgI* | NIF3 family metal-binding protein |
| b1015 | 77.17 | 73.17 | 29.06 | 0.38 | 0.40 | *putP* | proline:sodium symporter |
| b4058 | 239.90 | 308.71 | 122.96 | 0.51 | 0.40 | *uvrA* | ATPase and DNA damage recognition protein of nucleotide excision repair excinuclease UvrABC |
| b2208 | 19.48 | 26.38 | 10.56 | 0.54 | 0.40 | *napF* | ferredoxin-type protein |
| b1282 | 106.59 | 221.85 | 89.66 | 0.84 | 0.40 | *yciH* | initiation factor function partial mimic |
| b4085 | 17.72 | 20.77 | 8.43 | 0.48 | 0.41 | *alsE* | allulose-6-phosphate 3-epimerase |
| b0621 | 8.13 | 10.08 | 4.09 | 0.50 | 0.41 | *dcuC* | anaerobic C4-dicarboxylate transport |
| b1172 | 25.89 | 45.71 | 18.63 | 0.72 | 0.41 | *ymgG* | UPF0757 family protein |
| b4151 | 71.16 | 68.20 | 27.90 | 0.39 | 0.41 | *frdD* | fumarate reductase (anaerobic) |
| b0872 | 11.77 | 5.66 | 2.32 | 0.20 | 0.41 | *hcr* | HCP oxidoreductase |
| b1347 | 17.16 | 14.17 | 5.82 | 0.34 | 0.41 | *ydaC* | DUF1187 family protein |
| b0779 | 102.46 | 116.80 | 48.06 | 0.47 | 0.41 | *uvrB* | exision nuclease of nucleotide excision repair |
| b1223 | 22.15 | 25.23 | 10.46 | 0.47 | 0.41 | *narK* | nitrate/nitrite transporter |
| b2467 | 31.25 | 39.39 | 16.46 | 0.53 | 0.42 | *nudK* | GDP-mannose pyrophosphatase |
| b2617 | 249.73 | 359.73 | 150.96 | 0.60 | 0.42 | *bamE* | lipoprotein component of BamABCDE OM biogenesis complex |
| b3945 | 14.01 | 13.86 | 5.82 | 0.42 | 0.42 | *gldA* | glycerol dehydrogenase |
| b2240 | 165.23 | 138.41 | 58.29 | 0.35 | 0.42 | *glpT* | sn-glycerol-3-phosphate transporter |
| b0895 | 19.41 | 15.33 | 6.48 | 0.33 | 0.42 | *dmsB* | dimethyl sulfoxide reductase |
| b1004 | 9.24 | 14.41 | 6.10 | 0.66 | 0.42 | *wrbA* | NAD(P)H:quinone oxidoreductase |
| b2010 | 11.90 | 12.16 | 5.15 | 0.43 | 0.42 | *dacD* | D-alanyl-D-alanine carboxypeptidase |
| b1171 | 24.71 | 38.21 | 16.36 | 0.66 | 0.43 | *ymgD* | uncharacterized protein |
| b3267 | 32.24 | 40.85 | 17.50 | 0.54 | 0.43 | *yhdV* | putative outer membrane protein |
| b1664 | 40.81 | 41.29 | 17.69 | 0.43 | 0.43 | *ydhQ* | autotransporter adhesin-related protein |
| b2718 | 12.43 | 16.01 | 6.90 | 0.55 | 0.43 | *hycH* | hydrogenase 3 maturation protein |
| b0474 | 496.88 | 575.33 | 249.26 | 0.50 | 0.43 | *adk* | adenylate kinase |
| b2150 | 307.58 | 327.15 | 143.32 | 0.47 | 0.44 | *mglB* | methyl-galactoside transporter subunit |
| b2875 | 10.72 | 11.63 | 5.09 | 0.48 | 0.44 | *yqeB* | XdhC-CoxI family protein with NAD(P)-binding Rossman fold |
| b1739 | 12.53 | 18.75 | 8.22 | 0.66 | 0.44 | *osmE* | osmotically-inducible lipoprotein |
| b0200 | 52.61 | 63.08 | 27.70 | 0.53 | 0.44 | *gmhB* | D,D-heptose 1,7-bisphosphate phosphatase |
| b2313 | 201.20 | 145.25 | 63.91 | 0.32 | 0.44 | *cvpA* | colicin V production protein |
| b0817 | 40.31 | 42.47 | 18.69 | 0.46 | 0.44 | *mntR* | Mn(2+)-responsive manganese regulon transcriptional regulator |
| b4079 | 22.83 | 24.38 | 10.76 | 0.47 | 0.44 | *fdhF* | formate dehydrogenase-H |
| b4153 | 72.77 | 80.88 | 35.77 | 0.49 | 0.44 | *frdB* | fumarate reductase (anaerobic) |
| b2013 | 184.31 | 200.16 | 88.76 | 0.48 | 0.44 | *yeeE* | UPF0394 family inner membrane protein |
| b3103 | 11.93 | 19.85 | 8.81 | 0.74 | 0.44 | *yhaH* | DUF805 family inner membrane protein |
| b3510 | 15.19 | 29.97 | 13.35 | 0.88 | 0.45 | *hdeA* | stress response protein acid-resistance protein |
| b0231 | 36.92 | 48.27 | 21.52 | 0.58 | 0.45 | *dinB* | DNA polymerase IV |
| b2097 | 8.69 | 12.32 | 5.50 | 0.63 | 0.45 | *fbaB* | fructose-bisphosphate aldolase class I |
| b3707 | 951.32 | 992.11 | 443.38 | 0.47 | 0.45 | *tnaC* | tryptophanase leader peptide |
| b3959 | 10.04 | 11.67 | 5.22 | 0.52 | 0.45 | *argB* | acetylglutamate kinase |
| b1086 | 213.17 | 244.48 | 109.39 | 0.51 | 0.45 | *rluC* | 23S rRNA pseudouridine(955 |
| b0753 | 21.01 | 26.35 | 11.80 | 0.56 | 0.45 | *ybgS* | putative periplasmic protein |
| b4662 | 45.25 | 29.75 | 13.33 | 0.29 | 0.45 | *sgrT* | inhibitor of glucose uptake |
| b1444 | 9.82 | 13.52 | 6.07 | 0.62 | 0.45 | *patD* | gamma-aminobutyraldehyde dehydrogenase |
| b3124 | 12.06 | 10.72 | 4.82 | 0.40 | 0.45 | *garK* | glycerate kinase I |
| b4152 | 95.96 | 85.72 | 38.62 | 0.40 | 0.45 | *frdC* | fumarate reductase (anaerobic) |
| b1298 | 25.26 | 24.50 | 11.08 | 0.44 | 0.45 | *puuD* | gamma-glutamyl-gamma-aminobutyrate hydrolase |
| b0588 | 15.10 | 20.76 | 9.43 | 0.62 | 0.45 | *fepC* | ferrienterobactin ABC transporter ATPase |
| b1957 | 43.36 | 55.53 | 25.23 | 0.58 | 0.45 | *yodC* | uncharacterized protein |
| b1747 | 24.80 | 24.68 | 11.26 | 0.45 | 0.46 | *astA* | arginine succinyltransferase |
| b1348 | 11.28 | 12.35 | 5.64 | 0.50 | 0.46 | *ralR* | Rac prophage |
| b1992 | 13.39 | 12.05 | 5.52 | 0.41 | 0.46 | *cobS* | cobalamin synthase |
| b2614 | 180.07 | 230.87 | 106.12 | 0.59 | 0.46 | *grpE* | heat shock protein |
| b0220 | 18.45 | 27.07 | 12.53 | 0.68 | 0.46 | *ivy* | inhibitor of c-type lysozyme |
| b4545 | 8.96 | 10.32 | 4.79 | 0.53 | 0.46 | *ypdJ* |  |
| b1482 | 25.14 | 32.52 | 15.10 | 0.60 | 0.46 | *osmC* | lipoyl-dependent Cys-based peroxidase |
| b1779 | 1094.13 | 971.82 | 453.51 | 0.41 | 0.47 | *gapA* | glyceraldehyde-3-phosphate dehydrogenase A |
| b2239 | 345.44 | 290.79 | 135.71 | 0.39 | 0.47 | *glpQ* | periplasmic glycerophosphodiester phosphodiesterase |
| b2975 | 11.42 | 10.01 | 4.69 | 0.41 | 0.47 | *glcA* | glycolate transporter |
| b4577 | 27.58 | 19.26 | 9.04 | 0.33 | 0.47 | *sgrS* | sRNA antisense regulator destabilzes ptsG mRNA |
| b0831 | 17.29 | 14.24 | 6.71 | 0.39 | 0.47 | *gsiC* | glutathione ABC transporter permease |
| b2537 | 28.69 | 21.74 | 10.26 | 0.36 | 0.47 | *hcaR* | hca operon transcriptional regulator |
| b4154 | 60.56 | 65.38 | 30.86 | 0.51 | 0.47 | *frdA* | anaerobic fumarate reductase catalytic and NAD/flavoprotein subunit |
| b3917 | 94.17 | 180.99 | 85.74 | 0.91 | 0.47 | *sbp* | sulfate transporter subunit |
| b1746 | 18.43 | 18.31 | 8.69 | 0.47 | 0.47 | *astD* | succinylglutamic semialdehyde dehydrogenase |
| b2267 | 13.48 | 17.92 | 8.52 | 0.63 | 0.48 | *elaA* | GNAT family putative N-acetyltransferase |
| b4212 | 11.10 | 24.39 | 11.61 | 1.05 | 0.48 | *ytfH* | DUF24 family HxlR-type putative transcriptional regulator |
| b1189 | 64.59 | 58.92 | 28.14 | 0.44 | 0.48 | *dadA* | D-amino acid dehydrogenase |
| b0161 | 32.25 | 38.62 | 18.47 | 0.57 | 0.48 | *degP* | serine endoprotease (protease Do) |
| b4699 | 12.76 | 15.43 | 7.41 | 0.58 | 0.48 | *fnrS* | FNR-activated anaerobic sRNA antisense regulator down regulates cydDC |
| b0583 | 18.64 | 23.34 | 11.21 | 0.60 | 0.48 | *entD* | phosphopantetheinyltransferase component of enterobactin synthase multienzyme complex |
| b4198 | 12.73 | 16.35 | 7.90 | 0.62 | 0.48 | *ulaF* | L-ribulose 5-phosphate 4-epimerase |
| b1083 | 11.01 | 10.40 | 5.10 | 0.46 | 0.49 | *flgL* | flagellar hook-filament junction protein |
| b0382 | 16.01 | 25.75 | 12.64 | 0.79 | 0.49 | *iraP* | anti-RssB factor |
| b1770 | 18.41 | 18.30 | 8.98 | 0.49 | 0.49 | *ydjF* | putative DNA-binding transcriptional regulator |
| b4067 | 25.83 | 22.86 | 11.25 | 0.44 | 0.49 | *actP* | acetate transporter |
| b1533 | 22.39 | 23.80 | 11.73 | 0.52 | 0.49 | *eamA* | cysteine and O-acetyl-L-serine efflux system |
| b2200 | 16.97 | 21.07 | 10.43 | 0.61 | 0.50 | *ccmB* | heme export ABC transporter permease |
| b4457 | 377.27 | 367.65 | 182.50 | 0.48 | 0.50 | *csrC* | CsrC sRNA sequesters CsrA |
| b1745 | 20.13 | 18.16 | 9.02 | 0.45 | 0.50 | *astB* | succinylarginine dihydrolase |
| b4084 | 13.04 | 13.21 | 6.57 | 0.50 | 0.50 | *alsK* | D-allose kinase |
| b2201 | 54.85 | 55.83 | 27.76 | 0.51 | 0.50 | *ccmA* | heme export ABC transporter ATPase |
| b2207 | 14.48 | 19.71 | 9.81 | 0.68 | 0.50 | *napD* | assembly protein for periplasmic nitrate reductase |

^a^ List for the most differentially downregulated genes is ordered from lowest to highest according to the fold change (*ardC ^-^/ ardC^+^*) column.
