# Supplementary material for: ArdC, a ssDNA-binding protein with a metalloprotease domain, overpasses the recipient hsdRMS restriction system broadening conjugation host range
Source: PLoS Genet. 2020 Apr 29;16(4):e1008750. doi: 10.1371/journal.pgen.1008750 (PMC7213743; doi:10.1371/journal.pgen.1008750)
Supplement: S8 Table — (DOCX) [file pgen.1008750.s015.docx]

S8 Table. Expression profile of differentially upregulated *P. putida* genes in the presence/absence of ardC.

| **Locus tag** | **RPKMs** | | | **Fold Change** | | **Information** | |
| --- | --- | --- | --- | --- | --- | --- | --- |
|  | **NP** | ***ardC* +** | ***ardC -*** | ***ardC*+ / NP** | ***ardC +* / *ardC -* ^a^** | **Gene name^b^** | **Protein function** |
| PP_5744 | 17.85 | 76.29 | 15.54 | 4.27 | 4.91 | ***hp*** | hypothetical protein |
| PP_3773 | 6.83 | 50.81 | 13.37 | 7.44 | 3.80 |  | hypothetical protein |
| PP_4616 | 63.44 | 297.59 | 79.26 | 4.69 | 3.75 | ***yebG*** | hypothetical protein |
| PP_2177 | 8.80 | 28.72 | 7.82 | 3.26 | 3.68 |  | transcriptional regulator |
| PP_4729 | 19.59 | 76.16 | 21.35 | 3.89 | 3.57 | ***recN*** | DNA repair/recombination protein |
| PP_4617 | 20.34 | 107.73 | 30.50 | 5.30 | 3.53 | *ldh* | leucine dehydrogenase |
| PP_5580 | 41.92 | 33.37 | 9.66 | 0.80 | 3.45 |  | hypothetical protein |
| PP_2109 | 29.56 | 124.74 | 36.42 | 4.22 | 3.43 | ***hp*** | hypothetical protein |
| PP_2143 | 57.97 | 206.22 | 62.24 | 3.56 | 3.31 | ***lexA-I*** | transcriptional repressor |
| PP_3694 | 9.07 | 12.83 | 4.00 | 1.41 | 3.21 |  | hypothetical protein |
| PP_3850 | 4.66 | 15.37 | 4.88 | 3.30 | 3.15 |  | hypothetical protein |
| PP_2924 | 8.62 | 23.13 | 7.75 | 2.68 | 2.98 | ***hp*** | hypothetical protein |
| PP_5579 | 29.32 | 24.10 | 8.63 | 0.82 | 2.79 |  | hypothetical protein |
| PP_4346 | 24.89 | 25.20 | 9.12 | 1.01 | 2.76 | *ddlA* | D-alanine--D-alanine ligase A |
| PP_1521 | 101.19 | 107.05 | 39.51 | 1.06 | 2.71 |  | hypothetical protein |
| PP_5091 | 125.26 | 140.77 | 51.97 | 1.12 | 2.71 |  | membrane protein |
| PP_mr22 | 168.61 | 153.02 | 57.81 | 0.91 | 2.65 |  | small regulatory RNA RsmZ |
| PP_2451 | 44.65 | 121.59 | 47.33 | 2.72 | 2.57 | *endX* | extracellular DNA endonuclease |
| PP_1522 | 335.41 | 339.04 | 133.76 | 1.01 | 2.53 | *cspA-I* | major cold shock protein |
| PP_3109 | 13.62 | 9.23 | 3.66 | 0.68 | 2.52 |  | hypothetical protein |
| PP_5637 | 8.80 | 20.31 | 8.16 | 2.31 | 2.49 |  | hypothetical protein |
| PP_5487 | 177.81 | 210.85 | 84.80 | 1.19 | 2.49 | ***hp*** | hypothetical protein |
| PP_1203 | 20.96 | 57.26 | 24.19 | 2.73 | 2.37 | ***dinB*** | DNA polymerase IV |
| PP_1630 | 217.32 | 711.78 | 305.70 | 3.28 | 2.33 | ***recX*** | regulatory protein RecX |
| PP_3089 | 3217.68 | 4942.07 | 2134.61 | 1.54 | 2.32 | *hcp1* | Hcp1 |
| PP_3901 | 21.96 | 23.23 | 10.09 | 1.06 | 2.30 |  | hypothetical protein |
| PP_1629 | 462.05 | 1654.44 | 719.74 | 3.58 | 2.30 | ***recA*** | recombinase RecA |
| PP_1625 | 877.65 | 925.43 | 403.67 | 1.05 | 2.29 | *fdxA* | ferredoxin 1 |
| PP_2839 | 25.81 | 87.06 | 38.45 | 3.37 | 2.26 | ***hp*** | hypothetical protein |
| PP_2180 | 15.07 | 20.40 | 9.15 | 1.35 | 2.23 | *spuC-I* | polyamine:pyruvate transaminase |
| PP_4350 | 7.65 | 12.30 | 5.52 | 1.61 | 2.23 |  | class V NifS/IscS family aminotransferase |
| PP_5694 | 783.92 | 851.48 | 391.35 | 1.09 | 2.18 |  | hypothetical protein |
| PP_0641 | 244.70 | 480.50 | 221.45 | 1.96 | 2.17 |  | hypothetical protein |
| PP_4349 | 15.62 | 22.94 | 10.58 | 1.47 | 2.17 |  | hypothetical protein |
| PP_2838 | 46.94 | 143.77 | 66.48 | 3.06 | 2.16 |  | hypothetical protein |
| PP_5464 | 23.62 | 37.66 | 17.50 | 1.59 | 2.15 |  | hypothetical protein |
| PP_2840 | 11.19 | 32.80 | 15.53 | 2.93 | 2.11 |  | membrane protein |
| PP_3592 | 9.03 | 16.75 | 7.98 | 1.85 | 2.10 |  | RpiR family transcriptional regulator |
| PP_mr05 | 102.05 | 62.00 | 29.79 | 0.61 | 2.08 |  | small regulatory RNA RsmY |
| PP_1631 | 23.54 | 81.52 | 39.69 | 3.46 | 2.05 |  | hypothetical protein |
| PP_3117 | 61.71 | 159.22 | 78.42 | 2.58 | 2.03 | ***pp_3117*** | DNA lesion error-prone processing protein |
| PP_4856 | 15.70 | 23.01 | 11.43 | 1.47 | 2.01 |  | Dps family ferritin |
| PP_5554 | 68.41 | 38.93 | 19.46 | 0.57 | 2.00 |  | hypothetical protein |

^a^ Table is ordered from higher to lower values according to *ardC ^+^/ ardC ^-^ f*old changes column. ^b^ Gene names involved in the SOS signalling pathway are shown in bold.
