# Supplementary material for: ArdC, a ssDNA-binding protein with a metalloprotease domain, overpasses the recipient hsdRMS restriction system broadening conjugation host range
Source: PLoS Genet. 2020 Apr 29;16(4):e1008750. doi: 10.1371/journal.pgen.1008750 (PMC7213743; doi:10.1371/journal.pgen.1008750)
Supplement: S9 Table — (DOCX) [file pgen.1008750.s016.docx]

**Table S9. Oligonucleotides used in this study**

| **Oligonucleotide** | **Sequence (5’ - 3’) ^a^** |
| --- | --- |
| **ArdC-Nterm** | *TAGAAATAATTTTGTTTAACTTTAAGAAGGAGATATACAT*ATGAACGCAAAAACCAAGTTTGAC |
| **ArdC-Cterm** | *TAGCAGCCGGATCTCAGTGGTGGTGGTGGTGGTGCTCGAG*TGCGGCTTCTTTCCTTTGGA |
| **pET29CNdeI** | ATGTATATCTCCTTCTTAAAGTTAAAC |
| **pET29CXhoI** | CTCGAGCACCACCACC |
| **KfrAKamiK1** | *TCATACAACATACTACAGTACAGAGGCCCGCAAGAA*  *TGGCAATCACTAAA*GGTACCATCAAGAGACAGGATG  AGGATCGT |
| **Orf14KamiK2** | *GGAAAGGGCGGGTTCCCCCGCCCTCCCCTCGGTCA*  *AATGTGCGCGGCGGT*GGTACCAACCCCAGAGTCCC  GCTCAG |
| **N_Kn_Promoter_Wanner** | *AAATCAAAGCAGGCCCGGAAAAGCGCGGAAATGCAAGGGTTAAGCAGTGA*TACAGAGTTCTTGAAGTGGTGGCC |
| **C_Kn_Wanner** | *AACTGATGGCACAAAAAAAATCCCCCGCCGGAGCGGGGGAGGGCAGGTTA*GAAAAACTCATCGAGCATCAAATGA |
| **ArdC E229A d** | ATTGCCGATTTCTGCAATTAGT**G**CCTCGAAAGCGTAGCTCTTGCG |
| **ArdC E229A r** | CGCAAGAGCTACGCTTTCGAGG**C**ACTAATTGCAGAAATCGGCAAT |
| **ArdC_E229A_MAGE** | T*C*GTTTTAGCCGATTCAGCGACCGCAAGAGCTACGCTTTCGAGG**C**ACTAATTGCAGAAATCGGCAATTGCATGCTTTGCGCAAGCCTTGG |
| **ardC_rev(Hin)** | TGCAAGCTTTTATGCGGCTTCTTTCC |
| **ardC_fwd(Eco)** | GAGCTCGAATTCGTGACCCGGAACAAAG |
| **Up** | CGCTCCCTTCACTCGGAAATC |
| **Down** | CGAACGGCCCGGATTGA |
| **Middle Up** | GGGGATCGCAGTGGTGAGTAAC |
| **Middle Down** | CTTTTGCCATTCTCACCGGA |
| **T87I1 (45b)** | GAGCGCATCGGCCTTGACCTCATATTCAGCGCGCCCAAGAGCGTA |
| **T87I2 (45b)** | TACGCTCTTGGGCGCGCTGAATATGAGGTCAAGGCCGATGCGCTC |
| **Fluor-T87I2 (45b)** | 6FAM-TACGCTCTTGGGCGCGCTGAATATGAGGTCAAGGCCGATGCGCTC |
| **Mid1 (18b)** | GAGCGCATCGGCCTTGAC |
| **Mid2 (27b)** | CTCATATTCAGCGCGCCCAAGAGCGTA |

^a^ Oligonucleotide sequences. *: phosphorothioate bonds. Underlined: restriction enzyme recognition sequence. Italics: tails for recombineering. Bold: mutagenic introduction. 6FAM: 6-carboxyfluorescein label.
